# CircCLMP Suppresses Anti-Tumor Immunity by Inhibiting Activation of IRF3 and Interferon Response in Microsatellite Instability-high Endometrial Cancer

**DOI:** 10.7150/ijbs.125547

**Published:** 2026-01-14

**Authors:** Weijia Wen, Li Yuan, Peng Guo, Xueyuan Zhao, Linna Chen, Songlin Liu, Haolin Fan, Lin Lin, Yu Pan, Shiyi Chen, Yifang Xiao, Pan Liu, Dongyun Wang, Hongye Jiang, Wei Wang, Chunyu Zhang, Shuzhong Yao

**Affiliations:** 1 Department of Obstetrics and Gynecology, the First Affiliated Hospital, Sun Yat-sen University, Guangzhou, Guangdong, P.R. China.; 2 Guangdong Provincial Clinical Research Center for Obstetrical and Gynecological Diseases, Guangzhou, Guangdong, P.R. China.; 3 Department of Gynecology, Jinghong First People's Hospital, Xishuangbanna, Yunnan, P.R. China.

**Keywords:** microsatellite instability-high endometrial cancer (MSI EC), circCLMP, anti-tumor immunity, IRF3, interferon response

## Abstract

Immune checkpoint inhibitors have been proven effective for recurrent or metastatic cases of microsatellite instability-high (MSI) endometrial cancer (EC). However, drug resistance exists in a noticeable proportion of patients. Elucidating the underlying mechanisms would help develop new therapeutic strategies and benefit in improving patients' prognosis. Circular RNAs (circRNAs) are excellent biomarkers due to their stability and tissue specificity. Evidence has showed that circRNAs could mediate immune evasion in several types of malignancies. However, whether they regulate the immune response in MSI EC has not been explored. Here, based on the results of our former circRNA array, which identified the differentially-expressed circRNAs in MSI EC, we found that a circRNA, circCLMP, was negatively correlated with CD8^+^ T cell infiltration in MSI EC, and up-regulated in ICI-resistant MSI EC. *In vivo* assays showed that circCLMP could alter the anti-tumor immunity and promote tumor growth. Mechanistically, circCLMP shielded IRF3 from binding to TBK1, interfered with the phosphorylation and nuclear translocation of IRF3, thereby inhibiting the activation of interferon response, suppressing CD8^+^ T cell infiltration in the tumor environment, and eventually mediating immune evasion and promoting the progression of MSI tumors. Targeted knockdown of circCLMP combined with anti-PD-1 inhibitor treatment effectively enhanced the anti-tumor effects in the preclinical MSI EC PDX model. For the first time, our study reported an immunoregulating circRNA in MSI EC, which may provide insights into developing new biomarkers and therapeutic targets for overcoming immunotherapy resistance in MSI EC.

## Introduction

Endometrial cancer (EC) is the sixth most common malignant tumor in women 1. Approximately 7%-15% of early-staged cases and 40%-70% of advanced-staged cases of EC suffer from recurrence and metastasis, with a 5-year survival rate of 20%-25%, while the therapeutic regimens for recurrent and metastatic EC are still palliative 2,3. Based on molecular characteristics, EC has been classified into 4 subtypes: POLE-ε mutant (POLE-mut), microsatellite instability-high (MSI-H/MSI) or mismatch repair deficient (MMRd), no specific molecular profile (NSMP) or microsatellite stable (MSS), and p53-abnormal (p53_abn_) 4. The MSI subtype is believed to respond favorably to immune checkpoint inhibitors (ICIs) due to its high infiltration rate of CD8^+^ T cells. Consistently, anti-programmed cell death protein 1 (anti-PD-1)-based immunotherapy has showed favorable clinical efficacy among patients with MSI EC in recent trials 5-7. However, the response rate of PD-1 inhibitors in MSI EC was inferior to that in other malignancies with MSI status, and a noticeable proportion of MSI ECs manifest drug resistance, the reasons for which remain inexplicit 8-10. Therefore, it is an urgent need to elucidate the potential biological mechanisms for this phenomenon in the hope of improving the prognosis of recurrent and metastatic MSI EC patients.

Circular RNAs (circRNAs) are a distinct category of non-coding RNAs for their covalently closed-looped structure from back-splicing of precursor mRNAs. CircRNAs are suitable biomarkers for their stable existence and tissue specificity 11. Accumulating evidence indicated that circRNAs could exert multiple biological functions and even alter the anti-tumor immune response in several kinds of malignancies. In non-small cell lung cancer, hsa_circ_0082374 could inhibit the expression of PD-L1 and suppress the function of CD8^+^ T cells 12. In nasopharyngeal carcinoma, circBART2.2 could mediate tumor immune escape by binding to RIG-I 13. In colorectal cancer, hsa_circ_0015497 could boost the infiltration of regulatory T cells by inhibiting PGAM1, leading to resistance to CTLA-4 inhibitor-based immunotherapy 14. Nevertheless, whether circRNAs could regulate the anti-tumor immunity in MSI EC has been little focused on.

Interferon response is well known for its pathogen-eliminating power in innate immune response, yet in cancer immunology, it is also an essential ligament to cancer therapy. Researches have shown that interferon response is indispensable for cancer-specific antigen presentation and effector T cells activation 15,16. In the tumor immune microenvironment, interferon response is stimulative for enhancing the memory and effector function of CD8^+^ T cells 17,18, augmenting immune-mediated cancer cell apoptosis, and suppressive for the function of immunosuppressive cells 19,20. In melanoma and colorectal cancer, deficiency of interferon response significantly induces resistance to ICIs 21,22.

Interferon regulatory factor 3 (IRF3) is a key transcription factor in the interferon response 23. On the occasion of pathogen invasion, pattern recognition receptors (PRRs) encountered with exogenous nucleic acids will activate IRF3 and enable it to enter the nucleus and interact with interferon-stimulated response element (ISRE), leading to elevated transcription of interferon and interferon-stimulated genes (ISGs) 24,25. In MSI cancer cells, abnormal nucleic acids accumulation due to the deficiency of mismatch repair function also induces IRF3 activation and alters the anti-tumor immune response 26. In colorectal cancer, IRF3 inhibition subsequent to USP4 upregulation suppressed anti-tumor immune response by negatively regulating the interferon response and thereby accelerating tumor progression 27. However, the roles of interferon response and IRF3 in the cancer-specific immunity in MSI EC have never been studied.

In this study, we identified a candidate circRNA, circCLMP from our former circRNA array results 28, to be negatively correlated with CD8^+^ T cell infiltration in the immune microenvironment of MSI EC via weighted correlation network analysis (WGCNA). By *in vitro* and *in vivo* experiments, we found that circCLMP could suppress the interferon response and render MSI cancer cells resistant to immune-mediated apoptosis. Mechanistically, circCLMP shielded IRF3 from binding to TBK1 and hindered its phosphorylation and activation, which thereby inhibited the transcription of ISGs and suppressed the activation of interferon response, eventually resulting in the acceleration MSI tumor growth.

## Materials and Methods

### Human specimens

15 normal endometrium tissues and 203 endometrial cancer tissues used in the study, consisting of 66 MSI EC tissues and 137 MSS EC tissues, were collected at the First Affiliated Hospital of Sun Yat-sen University from May, 2023 to September, 2025. Thirty-six MSI EC samples with ICB treatment outcomes (19 were responders, and 17 were non-responders) have completed at least 6 months of follow-up. Patients offering normal endometrium tissues who underwent hysterectomy were pathologically confirmed without endometrial diseases. Patients offering endometrial cancer tissues were required reception of comprehensive staging laparotomy and receiving no chemotherapy, endocrinotherapy or radiotherapy prior to surgery. The molecular classification of each EC samples was confirmed by immunohistochemistry assay and next generation sequencing (NGS). Paraffin-embedded pathological slides were obtained from the Department of Pathology of our center. All the enrolled patients had signed informed consent. All conducted patient-related experiments complied with the Declaration of Helsinki. Approval of this study was obtained from the Ethical Review Committee of the First Affiliated Hospital of Sun Yat-sen University.

### Cell culture

Normal endometrial cells were isolated from normal endometrial tissues. HEK 293T were purchased from American Type Culture Collection. EC cell line Ishikawa (ISK) was purchased from European Collection of Authenticated Cell Cultures, ECACC. EC cell lines HEC-1A, KLE, AN3CA, MFE280 and murine colorectal cancer cell line MC38 were purchased from Chinese Academy of Sciences (Shanghai, China). MFE280 and AN3CA was cultured in MEM (Gibco, USA) supplemented with NEAA (Corning, USA) and 10% FBS (Gibco, USA). All other cell lines were cultured in DMEM supplemented with 10% FBS. An incubator with 5% CO_2_ at 37℃ was used to maintain cells. All cell lines were authenticated by STR analyses. The MMR status of KLE, MFE280, ISK, AN3CA and HEC-1A were verified by western blot detecting the expressions of 4 mismatch repair proteins.

### *In vivo* xenograft mice model

For xenograft mice experiment, we employed 6-8-week-old female Balb/c nude mice and C57BL/6J immunocompetent mice which were purchased from the Experimental Animal Center of Sun Yat-sen University. After being anesthetized with 1% pentobarbital sodium, each mouse within the same panel was inoculated subcutaneously with an equal amount of MC38 cells (3 × 10^6^ cells per mouse for cCLMP panels; 5 × 10^6^ cells per mouse for shcCLMP panels) mixed with Matrigel and PBS. Tumor volumes were measured and calculated as length × width^2^ × 0.52 every 10 days. On day 30 after tumor cell inoculation, mice were sacrificed and tumors were retrieved for weighing and subjected for paraffin-embedded slides. In preclinical CDX experiment, αPD-1 (Cat#HY-P99144, MCE, USA) of 200μg/injection/mouse or IgG isotype (Cat#HY-P990679, MCE, USA) of same dose was administered intraperitoneally twice a week until sacrifice, since 10 days after inoculation. For PDX model, fresh tumor tissues from patients who were pathologically diagnosed as MSI EC from preoperational biopsy were harvested from the First Affiliated Hospital of Sun Yat-sen University and immediately rinsed in PBS, suspended in DMEM supplemented with 100 U/mL penicillin/streptomycin, cut into pieces of 1-2 mm^3^, and then inoculated subcutaneously into the huPBMC-NCG mice (Gempharmatech, Guangdong, China) that had already be humanized by human PBMC and housed under SPF conditions. When the tumor size reached about 150mm^3^ (denoted as day 0), 5μl control or shcCLMP adeno-associated virus (AAV) (1×10^13^ v.g/ml), designed and synthesized by Obio Technology (Shanghai, China), were administered intratumorally for each mouse to generate the indicated groups. Three days after first AAV injection, a second dose of AAV injection of same procedure was administered. The procedure of αPD-1 or IgG administration was as mentioned above. The retrieved tumors were subjected to qRT-PCR for confirmation of transduction efficiency and to paraffin-embedded sections for CD8^+^ T cells evaluation. All the animal experiments were approved by the Animal Ethics Committee of Sun Yat-sen University (Approval number: SYSU-IACUC-2024-001966; Date: July 4^th^, 2024), and animals' care was in accordance with institution guidelines.

### T cell cytotoxic assay

T cells were isolated from C57BL/6J mouse spleen and human peripheral blood using magnetic bead separation kits according to the manufacturer's instructions (EasySep Direct Human T Cell Isolation Kit, Cat#19661, STEMCELL Technologies, Canada; EasySep Mouse Pan-Naïve T Cell Isolation Kit, Cat#19848, STEMCELL Technologies, Canada). T cells were activated by CD3/CD28 T cell activation beads (Cat#C0150, C0180, TargetMol, USA) for 7-10 days and cultured in RPMI 1640 (Gibco, USA) supplemented with IL-2 (10ng/ml, Cat#589102, 575402, BioLegend, USA). Then 2×10^4^ tumor cells of indicated groups were seeded on to 96-well plate and co-cultured with activated T cells in different Effector:Target (E:T) ratio. After 24h of incubation, the supernatants were subjected to LDH assay for cell lysis analysis. T cell cytotoxicity was calculated as: (sample OD490nm - background OD490nm)/(maximum OD490nm - background OD490nm).

### RNA extraction, reverse transcription, gDNA extraction, PCR, qRT-PCR, gel electrophoresis and cytoplasmic and nuclear RNA/protein isolation

The procedures of RNA extraction, reverse transcription and qRT-PCR were conducted as described previously. All primers used in this study were synthesized by GENEWIZ (Suzhou, China) and the primer sequences were listed in Table S1. gDNA extraction was conducted using FastPure Cell/Tissue DNA Isolation Mini Kit (Vazyme, Nanjing, China) and PCR was conducted using Phanta Max Super-Fidelity DNA Polymerase (Vazyme, Nanjing, China) following the manufacturer's instructions. For gel electrophoresis, we first prepare the gel with 1× TAE solution, 2% agarose and gel stain where we add into PCR products and DNA marker. Then the electrophoresis was conducted at a voltage of 120V for 15-30 minutes and photographed under ultraviolet light. Cytoplasmic and nuclear RNA/protein isolation was conducted using PARIS Kit (ThermoFisher, USA) according to the manufacturer's instructions. U6 and H3 were used as nuclear control and GAPDH and Cdr1as were used as cytoplasmic control.

### mRNA sequencing and data analysis

Total RNA was extracted using Trizol-based procedure. The RNA quality was check with the criteria of RIN > 7.0 before cDNA library construction. The VAHTS Universal V6 RNA-seq Library Prep Kit for Illumina (vazyme, Inc.) was used for cDNA library construction. In brief, mRNA containing poly-A tails was purified with oligo (dT) magnetic beads and fragmented into 200-600bp. The RNA fragments were subjected for cDNA synthesis, and then ligated with indexed adapters for purification. Second-strand cRNAs were removed with uracil glycosylase, and first-strand cDNA was for cDNA libraries construction. The libraries were sequenced by DNBSEQ-T7 on a 150 bp paired-end run. Clean reads were obtained and then aligned to human genome (GRCh38_Ensembl104) using the Star. HTseq was used to acquire gene counts. Differentially expressed genes were determined based on gene counts using DESeq2 R algorithm.

### Bioinformatic analysis

Gene ontology (GO) pathway enrichment analysis was performed via clusterProfile R package. *FDR/q* value less than 0.05 was considered significant. Gene set enrichment analysis (GSEA) was performed using the GSEABase R package and the enrichment scores and pathway visualization were obtained using the corrplot and ggplot2 R packages.

Weighted Correlation Network Analysis (WGCNA) was conducted using WGCNA R package where we set soft power as 20, TOMType as unsigned, minModuleSize as 25, corType as bicor, networkType as signed to generate the most suitable module division and expression matrix. The infiltration scores of 22 immune cells of the 10 sequenced MSI EC tissues were assessed via CIBERSORT (https://cibersortx.stanford.edu/) and served as the clinical traits in WGCNA to single out the CD8^+^ T cells infiltration-related module.

The circRNA structure prediction was performed using RNAfold WebServer tool based on the minimum free energy.

### Plasmid construction and transfection, lentivirus construction and transduction

Two different shRNA plasmids targeting the back-splicing junction (BSJ) of circCLMP were designed and synthesized by GenePharma (Suzhou, China) The sequences of shRNAs were provided in Table S2. For overexpressing circCLMP, we cloned the full length of circCLMP into lentivirus pLC5-Puro vector. The shcCLMP plasmid or cCLMP plasmid, along with psPAX2 and pMD2.G vectors, were cotransfected into lentiX-293T cells using X-tremeGENE HP DNA transfection reagent (Roche, Mannheim, Germany) to generate lentivirus particles which were harvested and filtered at 48h after transfection. Cells were then infected by lentivirus particles in complete medium containing 10 μg/ml Polybrene (Beyotime, Shanghai, China) and selected with puromycin (2 μg/ml, Sigma-Aldrich, USA) for 5 days.

### Single cell isolation and flow cytometry assay

To evaluate the immune cells infiltration of the *in vivo* xenograft mice model, single-cell suspension of immune cells from tumor tissues was acquired using a mouse Tumor Dissociation Kit (Cat# 130-096-730, Miltenyi Biotec, Germany) according to the manufacturer's protocol. After Fc blockade and viability assessment, 1×10^6^ cells of each condition were subjected to cell-surface marker staining in dark for 30 min and washed with diluent. Then the cells were fixed and permeabilized, followed by intracellular marker staining in dark for 20 min. The cells were analyzed using CytoFLEX (Beckman Coulter) and Flowjo software. The staining panels were provided in Table S3.

### RNase R assay and Actinomycin D assay

For RNase R treatment, we incubated cells with RNase R (Geneseed, Guangzhou, China) at 37 °C for 30 min. For actinomycin D assay, cells were seeded onto 6-well plate and treated with 2 mg/L actinomycin D (Sigma, USA) for 2h, 4h, 8h or 12h. Total RNAs of different groups were extracted and the levels of circCLMP and CLMP mRNA were detected by qRT-PCR.

### Immunohistochemistry (IHC), Immunofluorescence and immunoblotting

The procedures of IHC and CD8^+^ T cells infiltration IHC scores evaluation were conducted as described previously 28. For immunofluorescence of paraffin-embedded slides, we first deparaffinized and rehydrated the slides in xylene and graded ethanol. After antigen retrieval and sealing in goat serum, we incubated the slides with corresponding primary antibodies overnight under 4℃. After washing in PBS for 3 times, we incubated the slides with corresponding secondary antibodies conjugated with fluorescence for 1 h at room temperature. We then incubated the slides with DAPI to stain the nucleus and finally mounted with anti-fluorescence quenching mounting agent. An inverted fluorescence microscope was used to observe and photograph. For immunofluorescence of cell slide immunofluorescence, cells were seeded onto glass coverslips followed by fixation using 4% paraformaldehyde and incubation in 0.3% Triton X-100. The rest of procedures were as above. Immunoblot experiments were performed as described previously and the antibodies used in this study was detailed in Table S4.

### Cell proliferation assays

For CCK-8 assay, we added CCK-8 reagent (DOJINDO, Japan) into cells in 96-well plate according to manufacturer's protocol. After incubating for 2h, OD450nm values were detected. EdU assay was performed using Cell-Light EdU Apollo567 *In vitro* Kit (Ribobio, Guangzhou, China) according to manufacturer's protocol. A fluorescence microscope was used for image capturing. For colony formation assay, we seeded 1000 cells onto 6-well plates and cultured for 14 days before fixation and staining by crystal violet. Image J was used for data analysis.

### Lactate dehydrogenase (LDH) assay

LDH assay was performed using LDH Cytotoxicity Assay Kit (Beyotime, Shanghai, China) according to manufacturer's protocol. In brief, cells were seeded onto 96-well plates and treated by Poly (I:C) or diABZI (MedChemExpress, USA) for 8h. The culture supernatants were then incubated with LDH detection reagent for 30 min and the OD490nm values were detected.

### RNA pulldown assay and mass spectrometry

RNA pulldown assay was performed using Magnetic RNA-Protein Pull-Down Kit (ThermoFisher, USA) according to manufacturer's protocol. In brief, different truncations of circCLMP were amplified by PCR, transcribed into RNA using TranscriptAid T7 High Yield Transcription Kit (Thermo Fisher Scientific), labeled by biotin using RNA 3' End Biotinylation Kit (ThermoFisher, USA) and incubated with magnetic beads. Protein lysis was then added into the beads and incubated for 4h and washed with lysis buffer. The conjugated proteins were then separated and detected by western blotting or subjected to mass spectrometry (Fitgene Biotech, Guangzhou, China).

### RNA immunoprecipitation (RIP) assay and Co-immunoprecipitation

RIP assay was performed using Magna RIPTM RNA-binding protein immunoprecipitation kit (Millipore, USA). In brief, cell lysates were obtained with lysis buffer and then incubated with magnetic beads precoated with anti-IRF3 antibody or IgG at 4 ℃ overnight. After washing the beads, RNAs were purified with RNAiso plus (TaKaRa, Japan) and subjected to qRT-PCR. For co-IP assay, cell lysates were obtained with lysis buffer and then incubated with protein A/G magnetic beads (Cat#88802, ThermoFisher, USA) precoated with anti-IRF3 antibody or IgG at 4℃ overnight. After washing the beads, the precipitated proteins were subjected to western blotting.

### ISRE luciferase reporter assay

ISRE (interferon-stimulated response element) luciferase reporter assay was performed using Dual Luciferase Reporter Gene Assay Kit (Cat#RG027, Beyotime, China) according to manufacturer's protocol. In brief, cells in 96-well plates were co-transfected with pISRE-TA-luc (Cat#D2179, Beyotime, China) and pRL-TK (Cat#D2760, Beyotime, China). 100μL of reporter cell lysis buffer was added to each well for full lysis. After centrifugation, the supernatant was added into 100μL melted firefly luciferin enzyme reagent. The RLU (relative light unit) of each well was measured. Then 100μL melted renilla luciferin enzyme reagent was added to measure the RLU again. Reporter cell lysis buffer and luciferin enzyme reagents were used as blank control. Renilla luciferase activity was used for internal normalization and fold changes were calculated. ISRE luciferase activity was calculated by the formula: (sample firefly luminescence - background firefly luminescence)/(sample renilla luminescence - background renilla luminescence).

### Statistical analysis

SPSS 20.0 (SPSS Inc., Chicago, IL, USA) and GraphPad Prism 9.5 software were used for statistical analyses. Shapiro-Wilk method was used for normality test. Unpaired Student's t-test was used to compare the difference between two normality groups. Spearman's Rank Correlation analyses were used to determine correlation between circRNA expression and CD8 IHC scores. Chi-square test was used for clinicopathological parameters analysis.* P* < 0.05 was considered to be statistically significant.

## Results

### High circCLMP expression is negatively correlated with CD8^+^ T cell infiltration in MSI EC patients

**Figure 1:**
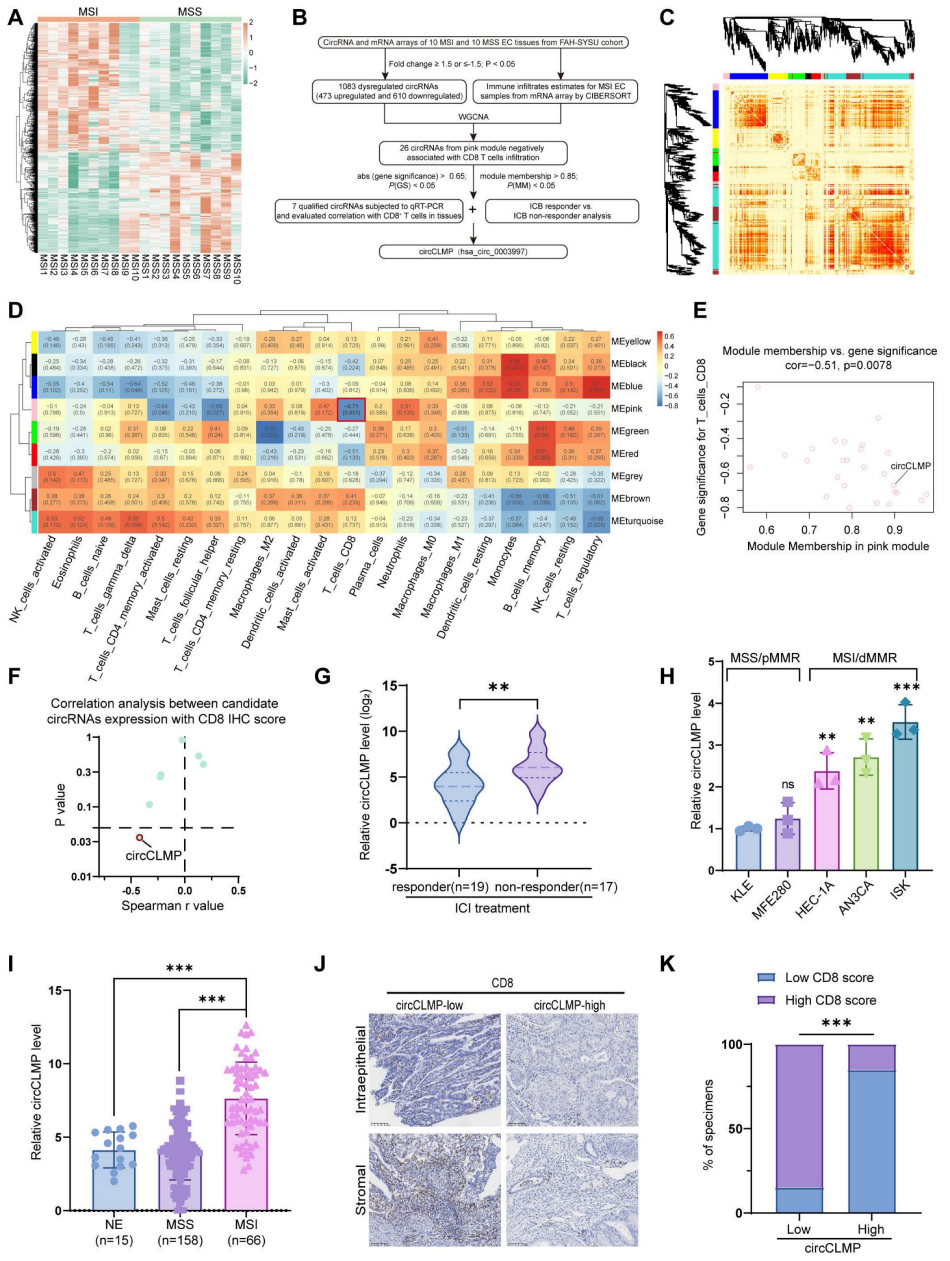
** circCLMP is negatively correlated to CD8^+^ T cells infiltration and up-regulated in ICB-resistant MSI EC patients. (A)** Heatmap demonstrated the differentially-expressed circRNAs between MSI EC and MSS EC from our former circRNA array analysis. Limma R package and Student's t test were used to determine DE-circRNAs with fold changes≥1.5 and p-value < 0.05 set as the cutoff values. **(B)** Flowchart of the steps utilized to identify and validate the MSI EC-related circRNA circCLMP. **(C)** The dendrogram and topological overlap matrix (TOM) sorted the differentially-expressed circRNAs in MSI EC into nine different modules from WGCNA R algorithm. **(D)** The correlation analysis between circRNA modules and immune cell infiltration estimates from WGCNA R algorithm. **(E)** Gene significance and module membership of each circRNA in pink module.** (F)** The Spearman correlation analyses between the expression level of 7 candidate circRNAs and CD8 IHC scores from another MSI EC cohort (n=25). **(G)** Recurrent or metastatic MSI EC patients not responding to ICB treatment exhibited higher circCLMP expression levels. Responder: n=19, non-responder: n=17. ACTB was used as internal reference. Student's t test was used for significance.** (H)** Relative circCLMP expression levels of several EC cell lines. **(I)** circCLMP expressions in clinical EC tissue samples in SYSU center. NE: n=15; MSS: n=158; MSI: n=66. Student's t test was used. **(I-J)** Representative images of CD8 staining and statistical analysis showing high circCLMP expression was accompanied by lesser CD8^+^ T cells infiltration. Original magnification, ×100. Chi-squared test was used for significance. Each experiment was performed at least three times independently. **P<0.01, ***P<0.001.

In our former study, we conducted circRNA array analysis to identify the differentially expressed circRNAs in MSI EC (Fig. 1A). To further screen out an MSI EC-related circRNA, we subjected the differentially expressed circRNAs to WGCNA, where nine circRNA modules were identified according to the circRNA expression pattern (Fig. 1B-C, Fig. S1A-D). We estimated the immune cells infiltration of the 10 sequenced MSI EC tissues by subjecting the mRNA sequencing data to the CIBERSORT algorithm (Fig. S1E). We selected the pink module for further screening from module-trait relationship analysis since it was significantly negatively correlated with CD8^+^ T cell infiltration and CD8^+^ T cells are the core effector cells in PD-1/PD-L1 inhibitor-based immunotherapy (Fig. 1D). Within the pink module, a criterion of module membership > 0.85, gene significance > -0.65, and *p* < 0.05 identified 7 potential circRNAs (Fig. 1E). Their potential negative correlation with CD8^+^ T cell infiltration was further verified in another EC cohort of our center using qRT-PCR and immunohistochemistry (IHC). The IHC score of CD8^+^ T cell infiltration was the sum of scores in the intraepithelial area and the stromal area of CD8-positive cells as previously described. Spearman correlation analysis showed that out of the 7 potential circRNAs, only circCLMP (hsa_circ_0003997) was significantly and negatively correlated with CD8^+^ T cell infiltration (Fig. 1F, Fig. S2A-G). Moreover, we detected the expression levels of these 7 candidate circRNAs in tissue specimens from MSI EC patients who received ICI treatments, and we found that only circCLMP was significantly upregulated in ICI non-responder EC (Fig. 1G, Fig. S3A-F). Next, we detected the expression levels of circCLMP in EC cell lines, whose MMR protein expressions were verified (Fig. S4) and consistent with previous literature 29. We found that circCLMP was significantly elevated in MSI/dMMR EC cell lines compared to MSS/pMMR EC cell lines (Fig. 1H). The circCLMP expressions in MSI EC tissues were also higher than those in MSS EC tissues and normal endometrium (NE) tissues (Fig. 1I). We also investigated the correlation between circCLMP expression and CD8^+^ T cell infiltration in a larger cohort of MSI EC patients. The results revealed that MSI EC patients with higher circCLMP expression levels exhibited milder CD8^+^ T cell infiltration (Fig. 1J,K). Next, we investigated the association between circCLMP expression and the clinicopathological characteristics of MSI EC patients and found that a higher level of circCLMP was significantly associated with higher FIGO grades, advanced FIGO stages, and larger tumor size of MSI EC (Table 1). Taken together, circCLMP was negatively correlated with CD8^+^ T cells infiltration and upregulated in ICI non-responder MSI EC patients. There findings suggested that circCLMP played a regulatory role in the anti-tumor immune response in MSI EC.

### Characteristics of circCLMP in MSI EC cells

**Figure 2:**
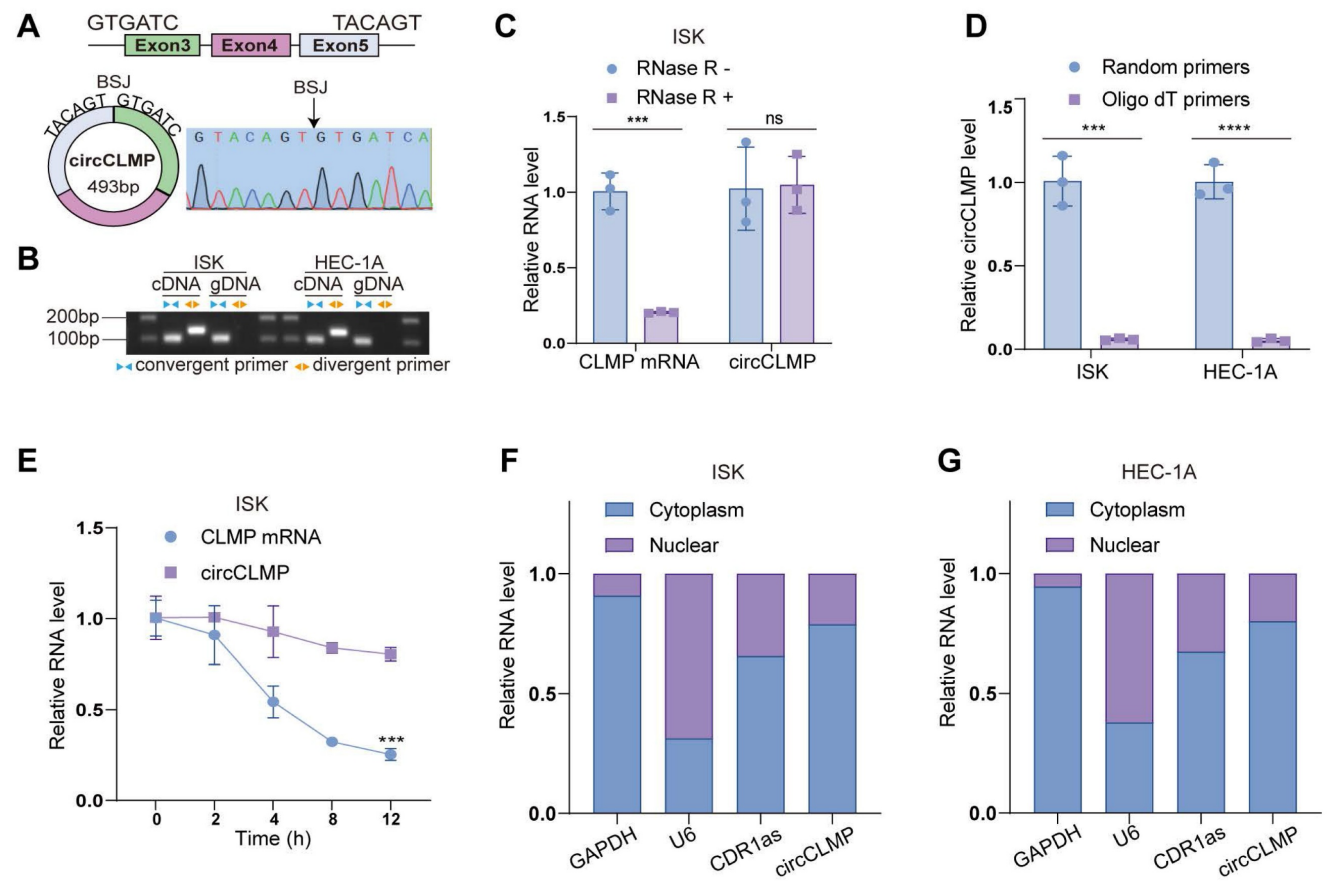
** Identifying the characteristics of circCLMP in EC cell lines. (A)** The genomic locus of circCLMP and the back splicing junction was identified by Sanger sequencing. **(B)** Gel electrophoresis showing the back splicing junction of circCLMP could only be amplified from cDNA using divergent primers. **(C)** qRT-qPCR analysis for the expression levels of circCLMP and CLMP mRNA in ISK treated with RNase R. **(D)** Random and oligo dT primers were used to detect the abundance of circCLMP in reverse transcription experiments. **(E)** qRT-qPCR analysis for the abundance of circCLMP and CLMP mRNA treated with actinomycin D at the indicated points. **(F-G)** Cytoplasmic and nuclear RNA isolation assay was conducted to detect the abundance of circCLMP in cytoplasm and nucleus. Each experiment was performed at least three times independently. Student's t test was used for significance. ns, no significant; ***P < 0.001; ****P < 0.0001.

Next, we used MSI EC cell lines, ISK and HEC-1A, for further validation of the characteristics of circCLMP. According to the annotation in circBase, circCLMP, 493 bp long, originates from the back-splicing of exon 3 and exon 5 of the CLMP mRNA, which was verified by Sanger sequencing (Fig. 2A). We amplified circCLMP with divergent primers targeting the BSJ and convergent primers targeting the linear counterpart in cDNA and gDNA and performed gel electrophoresis. The results showed that circCLMP was only amplified by divergent primers from cDNA but not from gDNA, which suggested that circCLMP was cyclized from its precursor mRNA (Fig. 2B). RNase R treatment broke down linear CLMP mRNA but not circCLMP (Fig. 2C, Fig. S5A). CircCLMP could only been reverse-transcribed using random primers but not oligo dT primers, proving the absence of a 3'-poly-adenylated tail (Fig. 2D). Under the treatment of actinomycin D, circCLMP showed a more stable quality than its linear counterpart (Fig. 2E, Fig. S5B). Additionally, qRT-PCR assay of cytoplasmic and nuclear fractions showed that circCLMP mainly existed in the cytoplasm (Fig. 2F, G). These results supported that circCLMP possessed a covalently closed looped structure.

### circCLMP promotes MSI-tumor growth by inhibiting anti-tumor immunity

**Figure 3:**
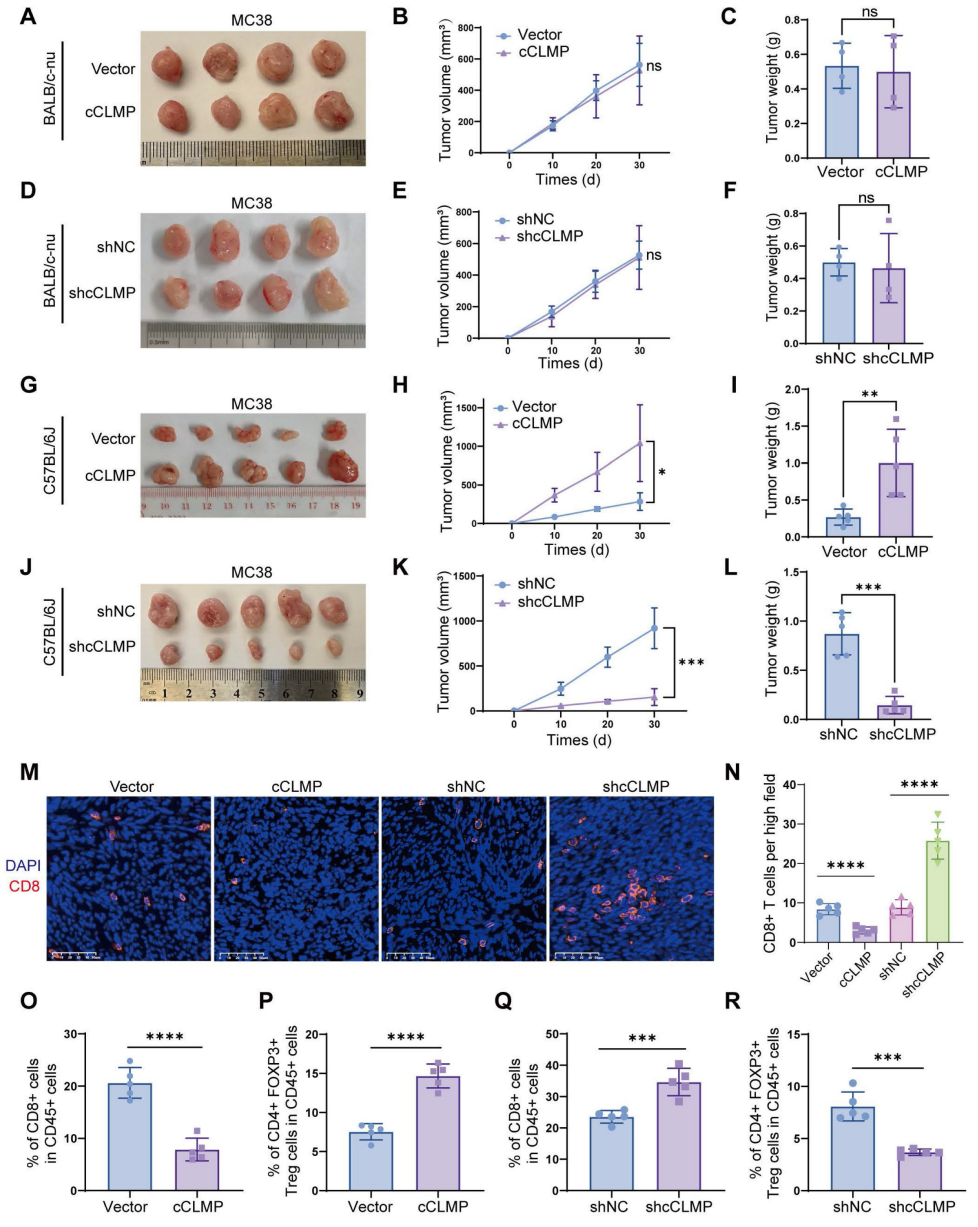
** CircCLMP promotes MSI-tumor growth by inhibiting anti-tumor immunity. (A-C)** Overexpressing circCLMP had no impact on MC38 tumor volumes or weights in BALB/c-nu mice (n=4 for each group). **(D-F)** Silencing circCLMP had no impact on MC38 tumor volumes or weights in BALB/c-nu mice (n=4 for each group). **(G-I)** In C57BL/6J immunocompetent mice, overexpressing circCLMP significantly boosted MC38 tumor volumes and weights (n=5 for each group), while **(J-L)** inhibiting circCLMP significantly decreased MC38 tumor volumes and weights (n=5 for each group). **(M-N)** Immunofluorescence assay showed CD8^+^ T cells infiltration of xenograft tumor was significantly decreased when circCLMP was overexpressed, but augmented when circCLMP was silenced. **(O-P)** Flow cytometry analysis showed that enhancing circCLMP expression significantly decreased CD8^+^ T cells proportion and elevated Treg cells ratio. **(Q-R)** Flow cytometry analysis showed that inhibiting circCLMP expression significantly enhanced CD8^+^ T cells proportion and lowered Treg cells ratio. Student's t test was used for significance. ns, no significant; *P<0.05; **P<0.01; ***P < 0.001; ****P < 0.0001.

Since circCLMP expression is positively correlated with tumor growth in MSI EC patients, we first carried out *in vitro* cell proliferation assays using MSI EC cell lines, ISK and HEC-1A. The efficiency of circCLMP expression manipulation was confirmed successful (Fig. S6A, B). CCK-8, EdU incorporation and colony formation assays demonstrated that the cell proliferation ability of ISK and HEC-1A was not affected by overexpression or depletion of circCLMP (Fig. S7A-H), suggesting that circCLMP might promote tumor growth by affecting anti-tumor immunity, but not by altering the proliferation ability. Since there is yet no available mouse MSI EC cell line, we utilized the mouse colorectal cancer cell lines MC38, which also exhibits MSI status, to conduct *in vivo* assays. We constructed circCLMP-knockdown, circCLMP-overexpressing and corresponding control MC38 cell lines (Fig. S6C, D) according to the highly conserved sequence as annotated in circBase, and grafted in BALB/c nude mice and immunocompetent mice C57BL/6J. Results showed that suppressing or overexpressing circCLMP did not alter the tumor growth in nude mice (Fig. 3A-F), but conversely in immunocompetent mice, circCLMP overexpression significantly promoted the tumor volumes and weights, while circCLMP knockdown significantly reduced tumor volumes and weights (Fig. 3G-L). Immunofluorescence assay showed that the CD8^+^ T cell infiltration in the tumor microenvironment significantly decreased upon forced expression of circCLMP, but increased when circCLMP expression was suppressed (Fig. 3M, N). Meanwhile, flow cytometry analysis of the grafted tumors showed that circCLMP overexpression markedly reduced CD8^+^ T cell infiltration but enhanced Treg cell infiltration. In contrast, silencing circCLMP exerted the opposite effects on CD8^+^ T/Treg cells ratio (Fig. 3O-R). To investigate whether circCLMP could alter the killing ability of CD8^+^ T cells, we isolated T cells from C57BL/6J spleen and human peripheral blood and activated them with CD3/CD28 activation beads and IL2. Corresponding tumor cells were co-cultured with activated T cells of different effector: target (E:T) ratios for 24 hours. Subsequently, cell lysis proportions were detected. We found that the T cell cytotoxicity was significantly augmented upon circCLMP inhibition, and impaired upon circCLMP overexpression (Fig. S8A-F). Taken together, these results revealed that circCLMP could affect the functions of tumor-infiltrating immune cells and inhibit anti-tumor immune response in MSI tumor models.

### CircCLMP suppresses IFN response and immune-mediated cell death in MSI EC cells

**Figure 4:**
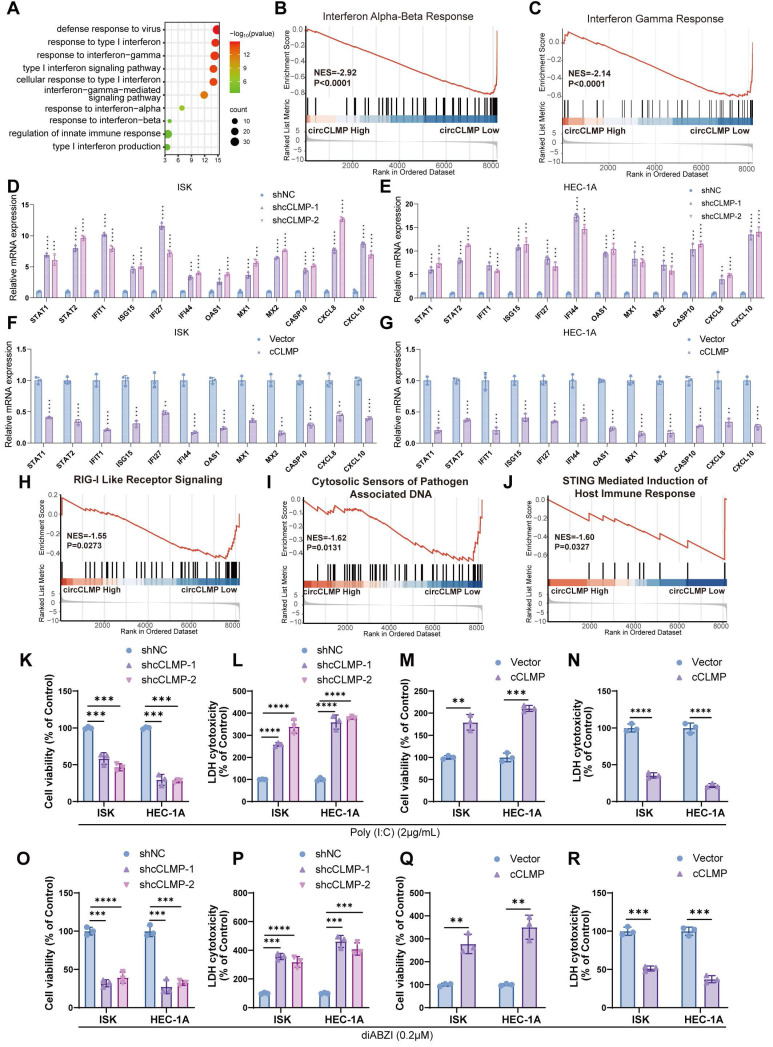
** circCLMP suppresses IFN response and immune-mediated cell death in MSI EC cells. (A)** GO analysis of the differentially expressed genes between ISK shNC cells and ISK shcCLMP cells enriched in interferon response and innate immunity. *FDR/q*<0.05 was considered significant. **(B-C)** GSEA analysis of the differentially expressed genes enriched in interferon response pathways. **(D-E)** Silencing circCLMP expressions in MSI EC cells significantly enhanced the ISGs expression. **(F-G)** Augmenting circCLMP expressions in MSI EC cells significantly inhibited the ISGs expression. **(H-J)** GSEA analyses showed PRR-mediated innate immune response pathways were enriched. **(K-L)** Under the treatment of poly (I:C), knockdown of circCLMP in ISK and HEC-1A significantly impaired cell viability and increased cell toxicity, while **(M-N)** overexpressing circCLMP in ISK and HEC-1A significantly increased cell viability and lowered cell toxicity. **(O-P)** Under the treatment of diABZI, knockdown of circCLMP in ISK and HEC-1A significantly impaired cell viability and increased cell toxicity, while **(Q-R)** overexpressing circCLMP in ISK and HEC-1A significantly increased cell viability and lowered cell toxicity. ACTB was used as internal reference. Each experiment was performed at least three times independently. Student's t test was used for significance between groups. **P<0.01; ***P < 0.001; ****P < 0.0001.

To further explore the biological processes regulated by circCLMP, we conducted transcriptomic sequencing in ISK cells. Gene ontology (GO) analysis of the differentially expressed genes altered by circCLMP knockdown revealed the enrichment of interferon (IFN) response pathways (Fig. 4A). Gene set enrichment analysis (GSEA) also demonstrated that IFN-α, IFN-β, and IFN-γ responses were activated in the circCLMP-low group (Fig. 4B, C). We then detected the expression levels of a series of interferon-stimulated genes (ISGs) that are usually upregulated during IFN response activation in cancer cells, including STAT1, STAT2, IFIT1, ISG15, IFI27, IFI44, OAS1, MX1, MX2, CASP10, CXCL8, and CXCL10. qRT-PCR revealed that downregulation of circCLMP significantly boosted the expressions of ISGs in ISK and HEC-1A (Fig. 4D, E), while overexpressing circCLMP significantly suppressed the expression levels of ISGs (Fig. 4F, G). Similar results were observed in MC38 cell lines (Fig. S9A, B). These results confirmed that circCLMP inhibited the activation of IFN response in MSI EC cells.

IFN response is an essential promoter in anti-tumor immunity and innate immunity. In most circumstances, IFN response is ignited when intracellular pattern recognition receptors (PRRs) sense aberrant nucleic acid particles, like exogenous DNA or RNA from pathogens and even endogenous ones from host cell itself 30. Among these PRRs, RIG-I-like receptors are the main molecular sensors for RNA, and cGAS-STING detects for DNA 31,32. Since our previous findings showed that circCLMP could inhibit the activation of IFN response, we wondered whether circCLMP regulates PRRs-related nucleic acid-sensing pathways. GSEA of the transcriptomic data indicated that suppression of circCLMP was associated with activation of RIG-I-like receptor signaling, cytosolic sensors of pathogen-associated DNA and STING-mediated induction of host immune response pathways (Fig. 4H-J).

To inspect the impact of circCLMP on RIG-I-like receptor signaling, we treated ISK and HEC-1A with poly (I:C), a dsRNA simulant and cell death inducer, and found that the cell viability was significantly impaired, and the LDH cytotoxicity increased when circCLMP was downregulated (Fig. 4K-L), while overexpression of circCLMP augmented the cell viability and reduced LDH cytotoxicity (Fig. 4M-N). For cytosolic sensors of pathogen-associated DNA pathway and STING-mediated induction of host immune response pathways, we adopted diABZI, a STING agonist and also cell death inducer, for verification. Under the condition of diABZI treatment, the cell death of ISK and HEC-1A cells was robustly enhanced when circCLMP was suppressed, but attenuated when circCLMP was elevated (Fig. 4O-R). Collectively, these results suggested that circCLMP suppressed IFN response and immune-mediated cell death in MSI EC cells.

### IRF3 is the downstream target of circCLMP

**Figure 5:**
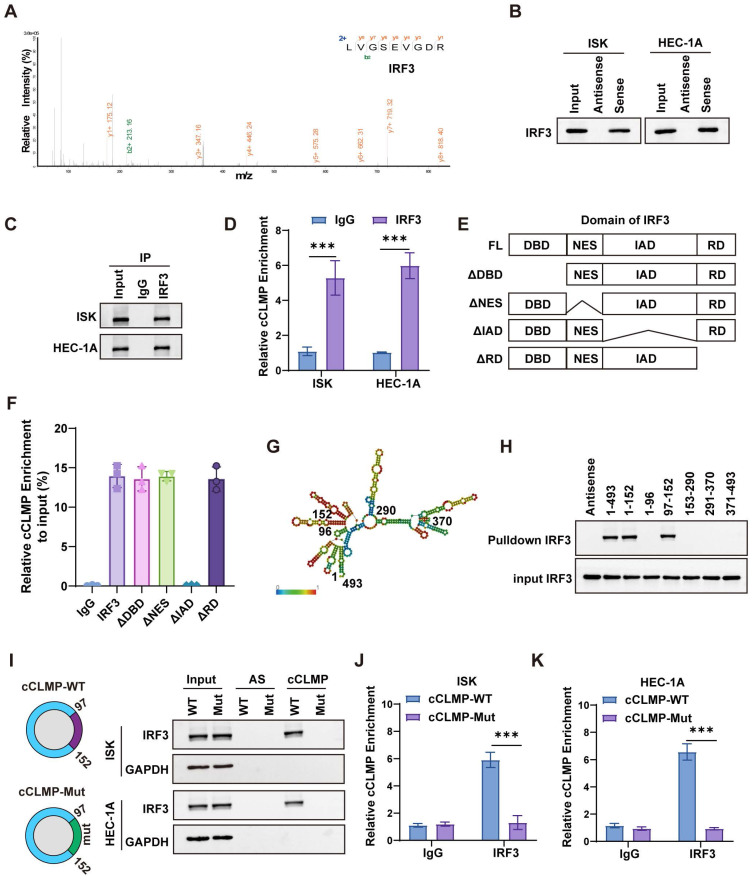
** IRF3 is the downstream target of circCLMP. (A)** Mass spectrometry identified IRF3 from the proteins bound to circCLMP. **(B)** RNA pulldown assay showed IRF3 was detected among proteins bound to circCLMP. **(C-D)** circCLMP could be detected among RNAs bound to IRF3 in RIP assay.** (E)** The four main domains of IRF3. **(F)** RIP assay showed that the IAD of IRF3 could bind to circCLMP. **(G)** The predicted structure of circCLMP. **(H)** RNA pulldown assay showed that the 97-152nt area of circCLMP could bind to IRF3. **(I)** IRF3 could be detected among proteins bound to wild type circCLMP but not circCLMP-Mut (97-152nt). **(J-K)** circCLMP-WT rather than circCLMP-Mut could be detected among RNAs bound to IRF3. Each experiment was performed at least three times independently. Student's t test was used for significance between groups. ***P < 0.001.

To further investigate the molecular mechanism by which circCLMP suppresses IFN response and anti-tumor immune response in MSI EC cells, we performed RNA pulldown and subjected the circCLMP-bound proteins to mass spectrometry analysis. We found that the abundance of interferon regulatory factor 3 (IRF3) ranked the top among the proteins binding to circCLMP (Fig, 5A, Table S5). Validation assays of the top 5 hits also suggested that IRF3 was the primary protein interacting with circCLMP (Fig. S10). IRF3 is a key transcription factor involved in the interferon response. Stably existing in the cytoplasm, IRF3 is phosphorylated and transferred into nucleus upon activation of the RIG-I pathway or cGAS-STING pathway. In the nucleus, IRF3 promotes the expression of IFN response genes by binding to IFN-stimulated response element (ISRE) 33. We further confirmed that circCLMP could bind to IRF3 in MSI EC cells with RNA pulldown and RNA immunoprecipitation (RIP) assays (Fig. 5B-D). IRF3 is mainly composed of DNA binding domain (DBD), nuclear export signal domain (NES), reaction domain (RD) and IRF associated domain (IAD). To clarify which exact domain of IRF3 could bind to circCLMP, we constructed truncated molecules of IRF3 and performed RIP-qPCR assay. Results revealed that the circCLMP mainly interacted with the IAD of IRF3 (Fig. 5E, F). Next, we predicted the structure of circCLMP based on minimum free energy and constructed truncated versions of circCLMP accordingly. RNA pulldown experiments showed that the 97-152 nt segment of circCLMP was the interacting motif with IRF3 (Fig. 5G, H). To determine whether the 97-152 nt motif contributes to IRF3 association, we then constructed the mutated version of circCLMP (97-152 nt) (cCLMP-Mut), and further pulldown assay and RIP assays confirmed that wild type of circCLMP (cCLMP-WT) rather than cCLMP-Mut could interact with IRF3 (Fig. 5I-K). These findings supported a direct interaction between IRF3 and the 97-152 nt region of circCLMP.

### CircCLMP restrains IRF3 activation and IFN transcription via blocking the TBK1 binding site on IRF3

**Figure 6:**
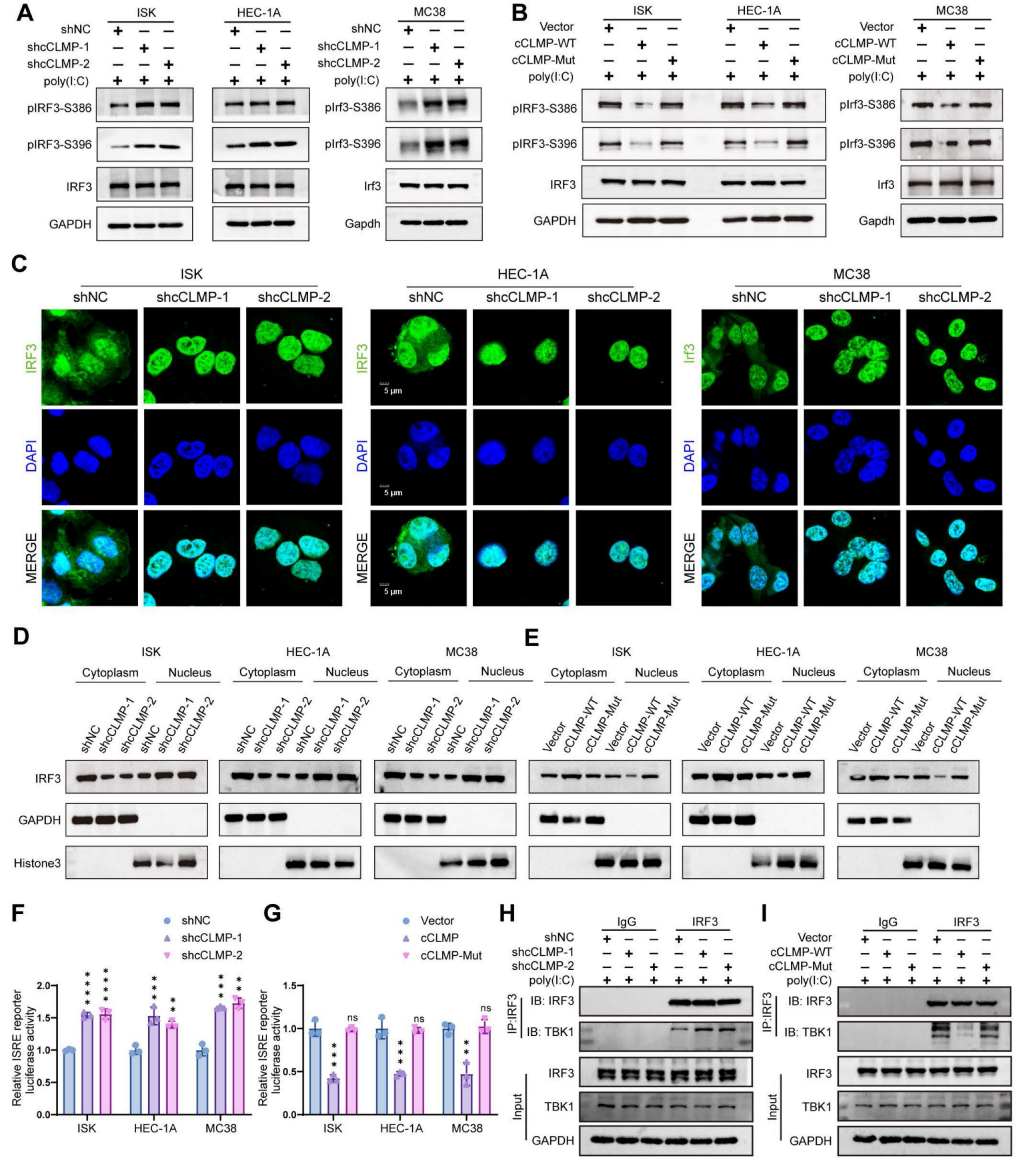
** circCLMP restrains IRF3 activation and IFN transcription via competing with TBK1 for IRF3 binding. (A)** Under the condition of poly (I:C), silencing circCLMP significantly increased the phosphorylation level of IRF3, while **(B)** overexpressing circCLMP-WT, rather than circCLMP-Mut, decreased the phosphorylation level of IRF3. **(C)** Immunofluorescence assay showed silencing circCLMP stimulated IRF3 nuclear translocation. **(D)** Cytoplasmic and nuclear isolation assay showed inhibiting circCLMP significantly decreased the abundance of IRF3 in cytoplasm and increased abundance in nucleus. **(E)** Overexpressing circCLMP, rather than circCLMP-Mut, significantly increased the proportion of IRF3 in cytoplasm and lowered the proportion of IRF3 in nucleus. **(F)** Silencing circCLMP significantly increased the promoter activity of ISRE. **(G)** Augmenting expression of circCLMP-WT, rather than circCLMP-Mut, significantly decreased ISRE reporter luciferase activity. **(H-I)** co-IP assay showed that under the treatment of poly (I:C), silencing circCLMP significantly increased the abundance of TBK1 bound to IRF3, while overexpressing circCLMP-WT, rather than circCLMP-Mut, significantly decreased the abundance of TBK1 bound to IRF3. Each experiment was performed at least three times independently. Student's t test was used for significance between groups. **P<0.01; ***P < 0.001; ****P < 0.0001.

Phosphorylation and nuclear translocation are indispensable for IRF3 activation. S386 and S396 are common phosphorylation sites for IRF3. Western blotting showed that circCLMP depletion significantly elevated the phosphorylation level of IRF3. Consistently, overexpression of cCLMP-WT rather than cCLMP-Mut decreased the phosphorylation level of IRF3 (Fig. 6A, B). Subsequently, we performed nuclear and cytoplasm fractionation assay and immunofluorescence assay and found that the nuclear translocation of IRF3 was enhanced upon depletion of circCLMP, and overexpressing cCLMP-WT but not cCLMP-Mut significantly restrained the nuclear translocation of IRF3 (Fig. 6C-E). Dual-luciferase assay using the ISRE luciferase reporter plasmid also showed that the promoter activity of the ISRE reporter was significantly augmented when circCLMP was suppressed, but inhibited upon cCLMP-WT overexpression and unaffected by cCLMP-Mut overexpression (Fig. 6F, G). These results suggested that the interaction between circCLMP and IRF3 impedes IRF3 activation and IFN transcription.

TANK binding kinase 1 (TBK1) is a well-established key upstream regulator responsible for IRF3 phosphorylation 34. It was reported that the C-terminal regulatory domain of IRF3, consisting of IAD and RD, is required for the phosphorylating function of TBK1 34,35. Therefore, it was hypothesized that the TBK1-mediated phosphorylation process of IRF3 was hindered by circCLMP. Co-immunoprecipitation (Co-IP) experiment showed that circCLMP knockdown significantly increased the binding level between TBK1 and IRF3, while overexpressing cCLMP-WT rather than cCLMP-Mut decreased the abundance of combined TBK1 on IRF3 (Fig. 6H, I), which suggested that circCLMP exerted its inhibiting function on IRF3 activation via shielding IRF3 from binding to TBK1.

### CircCLMP inhibits anti-tumor immunity in an IRF3-dependent manner

**Figure 7:**
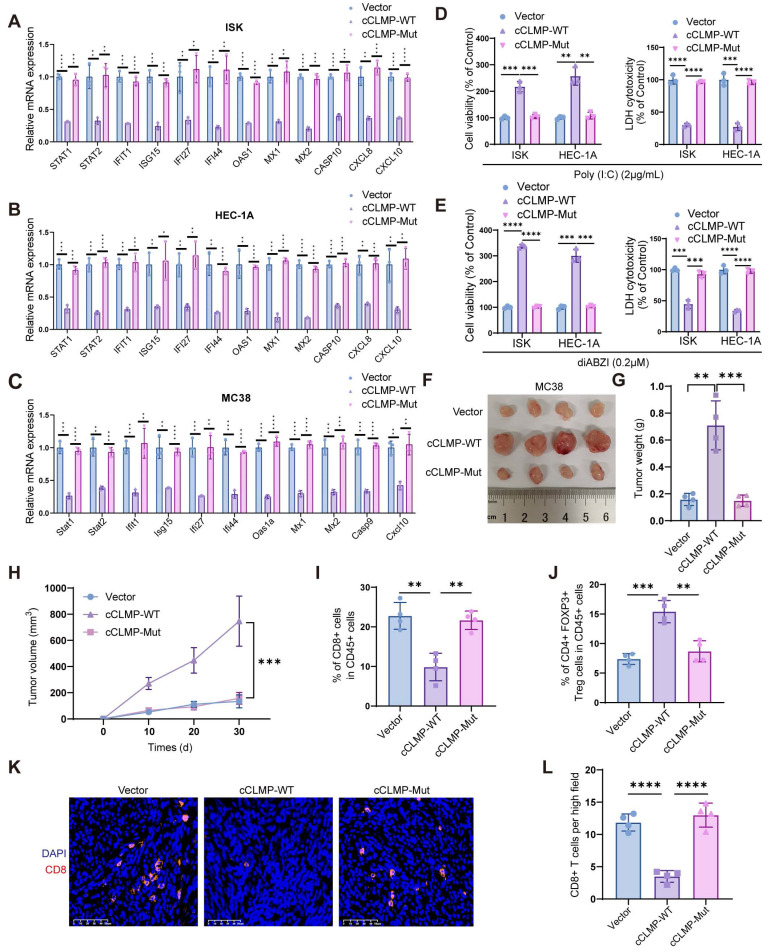
** circCLMP inhibits anti-tumor immunity in an IRF3-dependent manner. (A-C)** Overexpressing circCLMP-WT significantly decreased the expression of ISGs, while overexpressing circCLMP-Mut did not show such effect. **(D-E)** Under the condition of poly (I:C)/diABZI, overexpressing circCLMP significantly increased cell viability and lowered LDH cell toxicity, while overexpressing circCLMP-Mut showed no such effect. **(F-H)** Overexpressing circCLMP-WT, rather than circCLMP-Mut, significantly increased tumor volumes and weights (n=4 for each group). **(I-J)** Flow cytometry assay showed overexpressing circCLMP-WT, rather than circCLMP-Mut, significantly suppressed CD8^+^ T cells infiltration and elevated Treg cells ratio of xenograft tumors. **(K-L)** Immunofluorescence assay demonstrated that overexpressing circCLMP-WT significantly decreased CD8^+^ T cells infriltration, while overexpressing circCLMP-Mut showed no such effect. Student's t test was used for significance between groups. *P<0.05; **P<0.01; ***P < 0.001; ****P < 0.0001.

So as to figure out whether circCLMP inhibits the anti-tumor immunity in an IRF3-dependent manner, we performed a series of rescue assays. qRT-PCR experiments showed that overexpression of cCLMP-WT significantly lowered the expression of ISGs, while overexpressing cCLMP-Mut did not alter the expression of ISGs (Fig. 7A-C). The immune-mediated cell death caused by poly(I:C) and diABZI was significantly attenuated by elevated cCLMP-WT expression, but not affected by cCLMP-Mut overexpression (Fig. 7D, E). As expected, cCLMP-Mut failed to promote the tumor growth in *in vivo* xenograft experiments based on the findings of tumor volume and tumor weight (Fig. 7F-H). Moreover, cCLMP-WT overexpression led to significant decrease of CD8^+^ T cells infiltration and elevation of Treg ratio in the tumor immune environment when compared to the IRF3 interaction-deficient cCLMP-Mut overexpression (Fig. 7I, J). Furthermore, immunofluorescence assay also confirmed that cCLMP-WT overexpression led to a prominent decrease of the CD8^+^ T cell infiltration, while elevated cCLMP-Mut expression had no such effect (Fig. 7K, L). The impact of circCLMP on T cell cytotoxicity was also abrogated upon overexpressing cCLMP-Mut (Fig. S11). Taken together, these findings further supported the contribution of circCLMP-IRF3 interaction on inhibition of the anti-tumor immunity in MSI tumors.

### Combination strategy of targeting circCLMP and PD-1 blockade effectively inhibits MSI tumor growth in preclinical xenograft and PDX models

**Figure 8:**
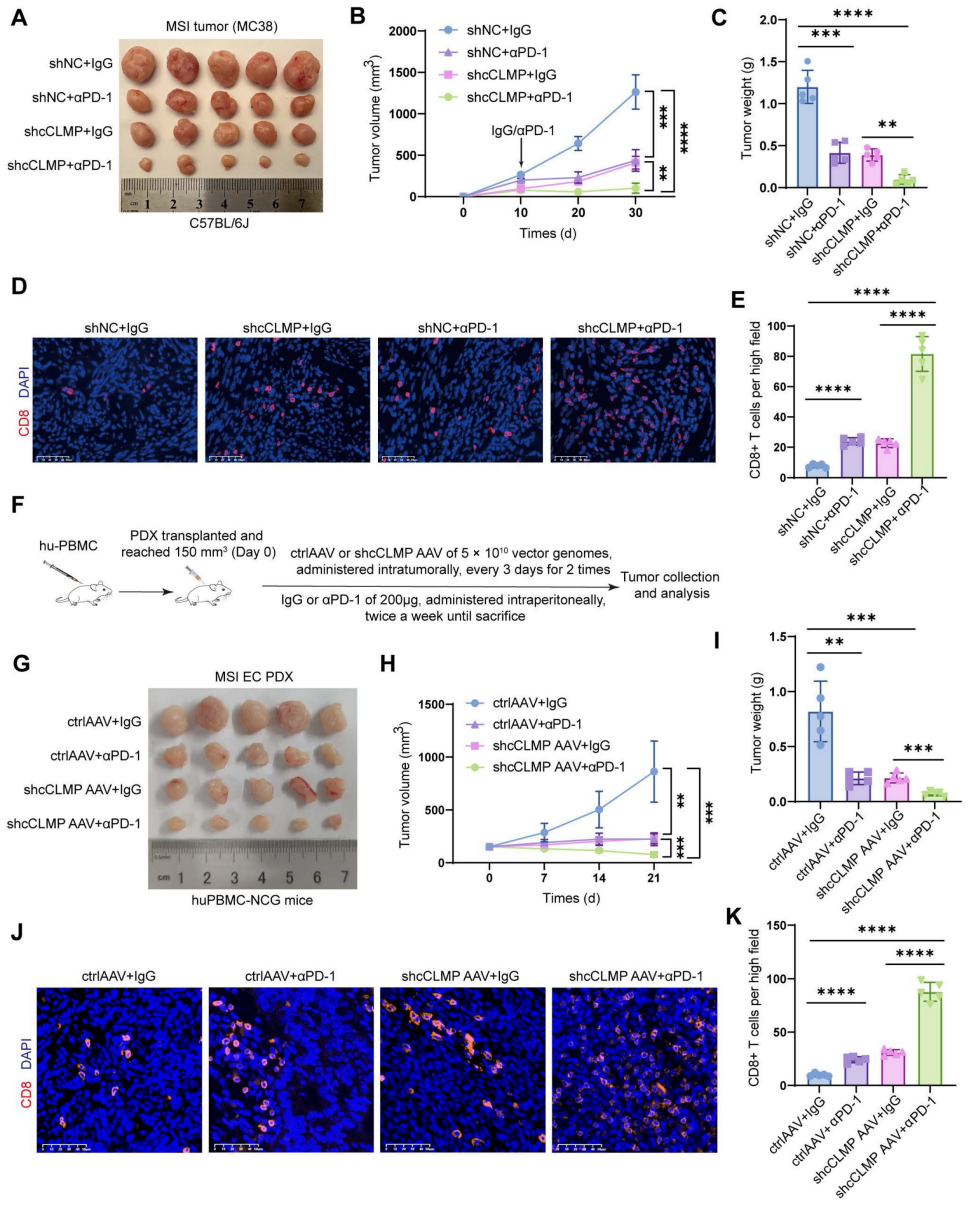
** Combination strategy of targeting circCLMP and blocking PD-1 efficiently impair MSI tumor growth in preclinical xenograft and PDX models. (A-C)** Targeting circCLMP combined with PD-1 inhibitor significantly suppresses tumor volumes and weights (n=5 for each group). **(D-E)** Targeting circCLMP combined with PD-1 inhibitor significantly increased CD8^+^ T cells infiltration as showed in immunofluorescence assay. **(F)** The workflow of MSI EC PDX model experiment. **(G-I)** In the MSI EC PDX model, combination strategy of targeting circCLMP and blocking PD-1 efficiently impair tumor volumes and tumor weights (n=5 for each group). **(J-K)** Immunofluorescence assay of the PDX xenograft model demonstrated that the CD8^+^ T cells were most abundant under treatment of targeting circCLMP and PD-1 blockade. Student's t test was used for significance between groups. **P < 0.01; ***P < 0.001; ****P < 0.0001.

Since the previous data indicated that circCLMP inhibited anti-tumor immunity and the CD8^+^ T cell infiltration in the tumor microenvironment, we deduced that knockdown of circCLMP may enhance the efficacy of PD-1 inhibitor-based therapy. Therefore, we conducted another panel of *in vivo* experiments using PD-1 inhibitor and confirmed that compared to circCLMP suppression or PD-1 inhibitor treatment alone, the combination of circCLMP knockdown and PD-1 inhibitor dramatically reduced the MSI tumor growth and augmented the CD8^+^ T cell infiltration in C57BL/6J mice (Fig. 8A-E). For the purpose of a condition more consistent to MSI EC, we established a humanized MSI-EC-PDX model on NCG mice by administering human peripheral blood mononuclear cells (hPBMCs) (Fig. 8F). Fresh MSI EC tissue pieces were grafted. The expression of circCLMP was suppressed using an adeno-associated virus vector (shcCLMP AAV) in formed tumors, and the control group received control AAV particles. The AAV transduction efficiency was verified by qPCR (Fig. S12). Similarly, in the humanized PDX model, the combination strategy of circCLMP depletion and PD-1 inhibitor markedly restrained the tumor growth and facilitated the CD8^+^ T cell infiltration (Fig. 8G-K). Taken together, our results indicated that the combination strategy of targeting circCLMP and PD-1 blockade contributed to a synergistic inhibitory effect for MSI tumors, and supported the potential of circCLMP as a therapeutic target for enhancing the efficacy of immunotherapy in MSI EC.

## Discussion

MSI status has been regarded as a marker for predicting satisfactory efficacy in cancer patients receiving immunotherapy 6. Among the four molecular subtypes of EC, MSI EC did show surpassing therapeutic response to ICIs than MSS EC. Yet, the response rate of ICI-based immunotherapy in MSI EC was much inferior to that in other cancer types of MSI status, such as MSI colorectal cancer 36,37. Drug resistance to ICIs exists noticeably in MSI EC. Although some may explain this phenomenon by the heterogeneity in tumor mutation burden, abundance of tumor-infiltrating immune cells, or the molecular initiators leading to the MSI condition within MSI EC, the exact molecular mechanisms remain blurry 38-40. Elucidating this uncertainty may help identify the proper candidates for ICIs treatment and overcome immunotherapy resistance in patients with MSI EC. With the development of high-throughput sequencing technologies, circRNAs have received much attention in the past few years and have been found to play important roles in cancer progression. Some circRNAs could interfere with anti-tumor immunity to facilitate cancer immune evasion. For instance, the m^6^A modification of circIGF2BP3 was found to inhibit CD8^+^ T cell responses and facilitate tumor immune evasion by promoting the deubiquitination of PD-L1 in non-small cell lung cancer 41. However, the functions of circRNAs in the anti-tumor immunity of MSI EC have not been reported. In our study, we identified an MSI EC-relevant circRNA circCLMP to be negatively associated with anti-tumor immunity for the first time. We confirmed the elevated expression of circCLMP in MSI EC tissues and cell lines as compared to MSS EC tissues and cell lines. We demonstrated that the ectopic upregulation of circCLMP was correlated with higher FIGO grades, more advanced FIGO stages, and larger tumor sizes in MSI EC. Moreover, our experimental data showed that circCLMP facilitated resistance to immune-mediated cell death *in vitro* and promoted tumor progression *in vivo*. Targeting circCLMP significantly enhanced the anti-tumor effects of PD-1 inhibitor in preclinical models. These findings reveal the suppressive role of circCLMP in the anti-tumor immunity of MSI EC and suggest the importance of circCLMP as a potential predictive biomarker and therapeutic target for immunotherapy resistance in MSI EC.

IFN response was first identified as an immune defense mechanism of host cells against bacteria and viruses. Yet it has been found that IFN response is also involved in anti-tumor immunity. The initiation of anti-tumor immunity requires the dendritic cells identifying and presenting cancer-specific antigens, and the antigen presentation process of dendritic cells relies on the support of IFN 15. In the presence of IFN, dendritic cells boost their secretion of immune stimulating factors, such as IL-12, IL-15, and tumor necrosis factors, which are necessary for the activation of tumor-specific CD8^+^ cytotoxic T cells 42,43. Studies have also revealed that IFN response could promote the memory function and cytotoxic function of CD8^+^ T cells, thereby augmenting immune-mediated cancer cell death 18. Moreover, IFN response could suppress the functions of inhibitory immune cells. For example, the insufficiency of IFN response could mediate cancer immune tolerance and poor prognosis in breast cancer by enhancing the infiltration of CD4^+^CD25^+^FOXP3^+^ regulatory T cells in the tumor microenvironment 19.

In the preclinical model of melanoma and colorectal cancer, the deficiency of IFN response was markedly correlated with the drug resistance of ICIs targeting the inhibitory receptors of CD8^+^ T cells, like CTLA-4 and PD-1 21,22. Accumulating evidence has indicated that the functioning IFN response is indispensable for an effective anti-tumor immunity. In this study, through mRNA sequencing, GO and GSEA analysis, we found that circCLMP suppressed the activation of IFN response in MSI EC cells. And by *in vitro* and *in vivo* assays, we demonstrated that circCLMP suppressed immune-mediated cancer cell death and CD8^+^ T cell infiltration in MSI tumors by inhibiting IFN response. Our findings support the essential role of a novel circCLMP-mediated IFN response mechanism in MSI EC.

IRF3, as a member of the interferon regulatory factor family, is a key transcription factor mediating the activation of IFN response. IRF3 is usually activated by the upstream molecules RIG-I/MDA5, which recognizes dsRNA, or by cGAS-STING, which recognizes DNA, and then interacts with ISRE, initiating the transcription of IFNs. The activation of IRF3 is significantly correlated with T cell response and cancer immunotherapy. In mice depleted of IRF3, the production of T cells and GZMB was markedly impaired 44. In cancer cells exposed to DNA damage response drugs and ICIs, IRF3 would get activated by cGAS-STING and then enhance the release of CXCL10 and CCL5 to augment the function of CD8^+^ T cells 45,46. In colorectal cancer, USP4 could suppress anti-tumor immunity by inhibiting IRF3 activation and tumor cell-intrinsic IFN response 27. The IRF3 pathway could also be mediated by non-coding RNAs. For example, a lncRNA BMOR, elevated in brain tissues, was shown to interact with IRF3 and impede its activation to facilitate metastatic colonization 47. In MSI tumors, it was reported that the deficiency of mismatch repair function would cause the accumulation of abnormal intracellular DNA, thereby leading to the aberrant activation of cGAS-STING-IRF3 pathway 26. However, the regulatory network underlying the role of IRF3 in MSI EC has not been fully understood. Herein, by RNA pulldown and co-IP assays, we revealed that circCLMP could bind to IRF3 and impede the TBK1-mediated phosphorylation and activation of IRF3, which led to cancer immune escape due to the insufficiency of anti-tumor immunity. Our findings highlight a novel circCLMP-mediated IRF3 inactivation mechanism in the immunotherapy resistance of MSI EC.

Since circCLMP exhibited prominent effect on suppressing anti-tumor immune response in MSI tumors, we constructed MSI tumor cell line and MSI EC PDX preclinical models to investigate the potential of circCLMP in the clinical treatment of recurrent or metastatic MSI tumors with ICIs. PDX models using humanized mice bearing patient-derived cancer tissues characterize a more consistent texture of intratumor heterogeneity and tumor microenvironment to real-world situations, making it an ideal choice for preclinical models 48. Among all types of virus particles manipulating gene expression, AAV seemed to be the best choice for clinical therapy because of its broad tissue tropism, poor immunogenicity, low cytotoxicity, and long-term stable transgene expression 49. AAV has been tried in gene therapy for certain diseases, and clinical trials have shown the safety and promising clinical applications of AAV for low vector-induced host immune response 50,51. Therefore, we employed AAV intratumoral administration to silence circCLMP expression. Our cell line and PDX xenograft data both demonstrated that the combination strategy of targeting circCLMP and PD-1 blockade treatment prominently suppressed MSI tumor growth and established a more vigorous tumor immune microenvironment, which serves as a piece of valid supplementary preclinical evidence of circCLMP being a promising target for overcoming ICI drug resistance for recurrent or metastatic MSI tumors.

However, certain limitations lie within our study. First, despite the efforts to elucidate the functions and downstream signaling pathways of circCLMP, the mechanisms of its upregulation in MSI EC was barely investigated. Second, the involved MSI EC patients receiving ICI treatments in our center were enrolled in the last two years. The survival data of these patients to support the predictive value of circCLMP necessitates long-term follow-up in the future research. And all patient samples came from our center, which may have induced potential geographic or selection bias. Collaboration with other centers in the future research may enhance the universality of our findings. Third, the lack of an available mouse MSI EC cell line makes it a second option to use MC38 for the *in vivo* immunocompetent studies. Although similar phenotypes were observed, cross-cancer immune-ecology differences still may have included confounding effects and raised potential concerns. Therefore, a PDX model was carried out for remedial *in vivo* evidence specific to MSI EC. A genetically engineered EC mouse model is warranted in the future research for more potent *in vivo* data.

**Figure 9:**
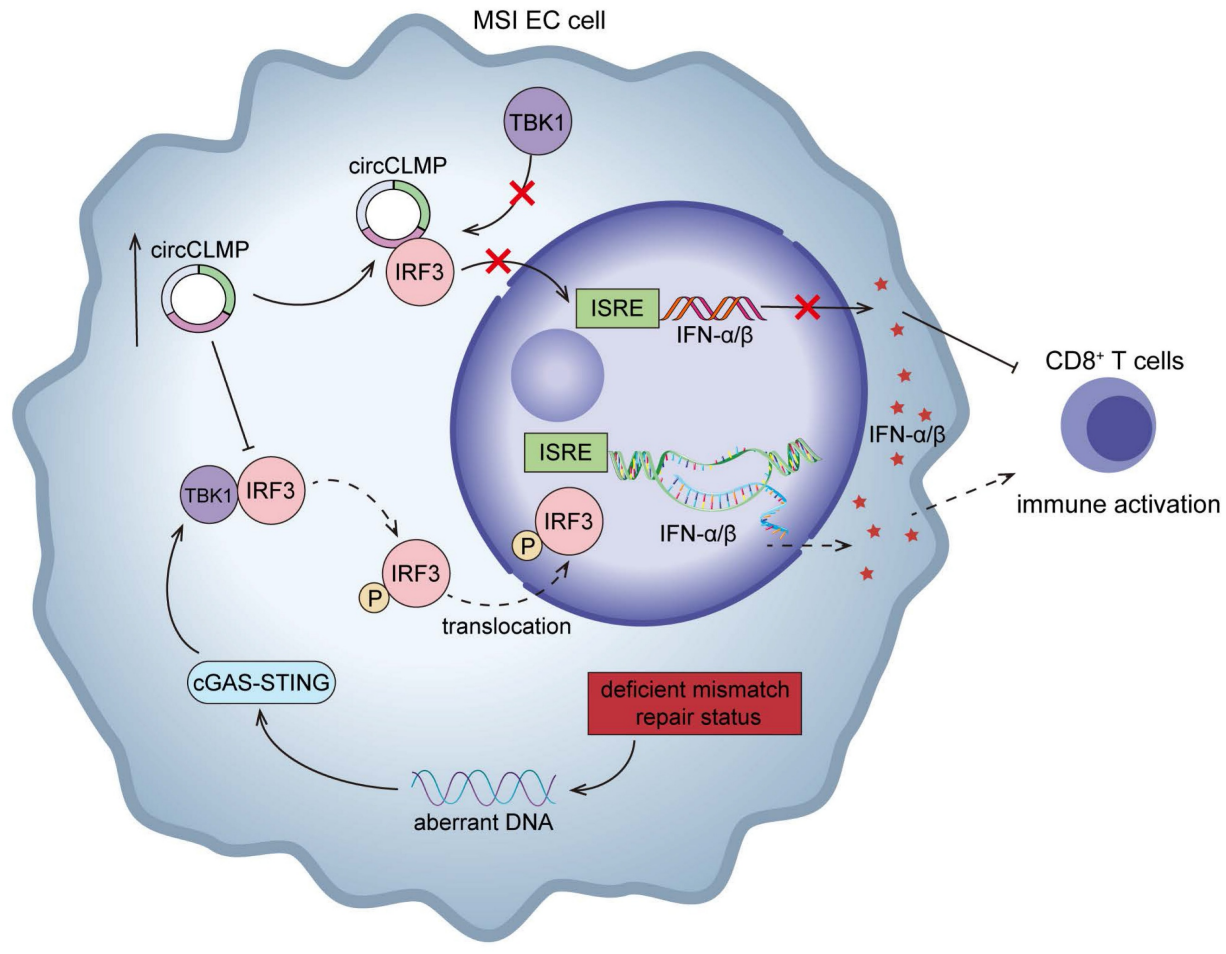
** Schematic illustration showing the mechanisms by which circCLMP suppresses anti-tumor immunity in MSI EC.** In MSI EC, deficient mismatch repair function causes aberrant activation of cGAS-STING pathway. Elevated circCLMP binds to cytoplasmic IRF3 and impedes its TBK1-mediated phosphorylation and nuclear translocation, leading to deficiency of ISRE promoter activity and downstream interferon response, which ultimately suppresses CD8^+^ T cells infiltration and facilitates tumor immune escape.

In conclusion, our study identifies a new circRNA, circCLMP, to negatively regulate anti-tumor immunity in MSI EC. Mechanistically, circCLMP shields the TBK1 binding site on IRF3, impeding the activation of IRF3 and downstream IFN response (Fig. 9). Combination of targeting circCLMP and PD-1 blockade showed favorable anti-tumor effects in preclinical models. In addition to providing new insights into the molecular mechanisms underlying immunotherapy resistance in MSI EC, our findings also propose a promising target for combination strategy with ICIs in MSI EC patients.

## Supplementary Material

Supplementary figures and tables.

## Figures and Tables

**Table 1:** Association between circCLMP expression and clinicopathological characteristics in MSI EC patients.

Clinicopathological characteristics	Total (N = 66)	circCLMP expression		P value
		Low	High	
Age/years				0.7783
< 60	49	25	24	
≥ 60	17	8	9	
BMI				0.6210
< 25	30	16	14	
≥ 25	36	17	19	
Tumor grade ^a^				**0.0174**
G1+G2	45	27	18	
G3	21	6	15	
Myometrial invasion				0.0825
< 1/2	37	22	15	
≥ 1/2	29	11	18	
LVSI				0.7997
Yes	25	13	12	
No	41	20	21	
FIGO stage				**0.0226**
I+II	54	31	23	
III+IV	12	2	10	
Tumor size				**0.0008**
< 2cm	23	18	5	
≥ 2cm	43	15	28	
Cervical stromal invasion				> 0.9999
Yes	5	3	2	
No	61	30	31	
Parametrial invasion				> 0.9999
Yes	1	0	1	
No	65	33	32	
Appendages metastasis ^b^				> 0.9999
Yes	3	2	1	
No	63	31	32	
Lymph node metastasis				0.1850
Yes	11	3	8	
No	55	30	25	
CD8^+^ T cells infiltration				**< 0.0001**
Low	33	5	28	
High	33	28	5	

a. Special pathological types were considered as G3. b. Appendages involvements of IA3 stage were excluded.
