# Supplementary material for: CircCLMP Suppresses Anti-Tumor Immunity by Inhibiting Activation of IRF3 and Interferon Response in Microsatellite Instability-high Endometrial Cancer
Source: Int J Biol Sci. 2026 Jan 14;22(3):1611–31. doi: 10.7150/ijbs.125547 (PMC12839119; doi:10.7150/ijbs.125547)
Supplement: Supplementary file 1 — Supplementary figures and tables. [file ijbsv22p1611s1.pdf]

Supplementary Figures

Figure S1

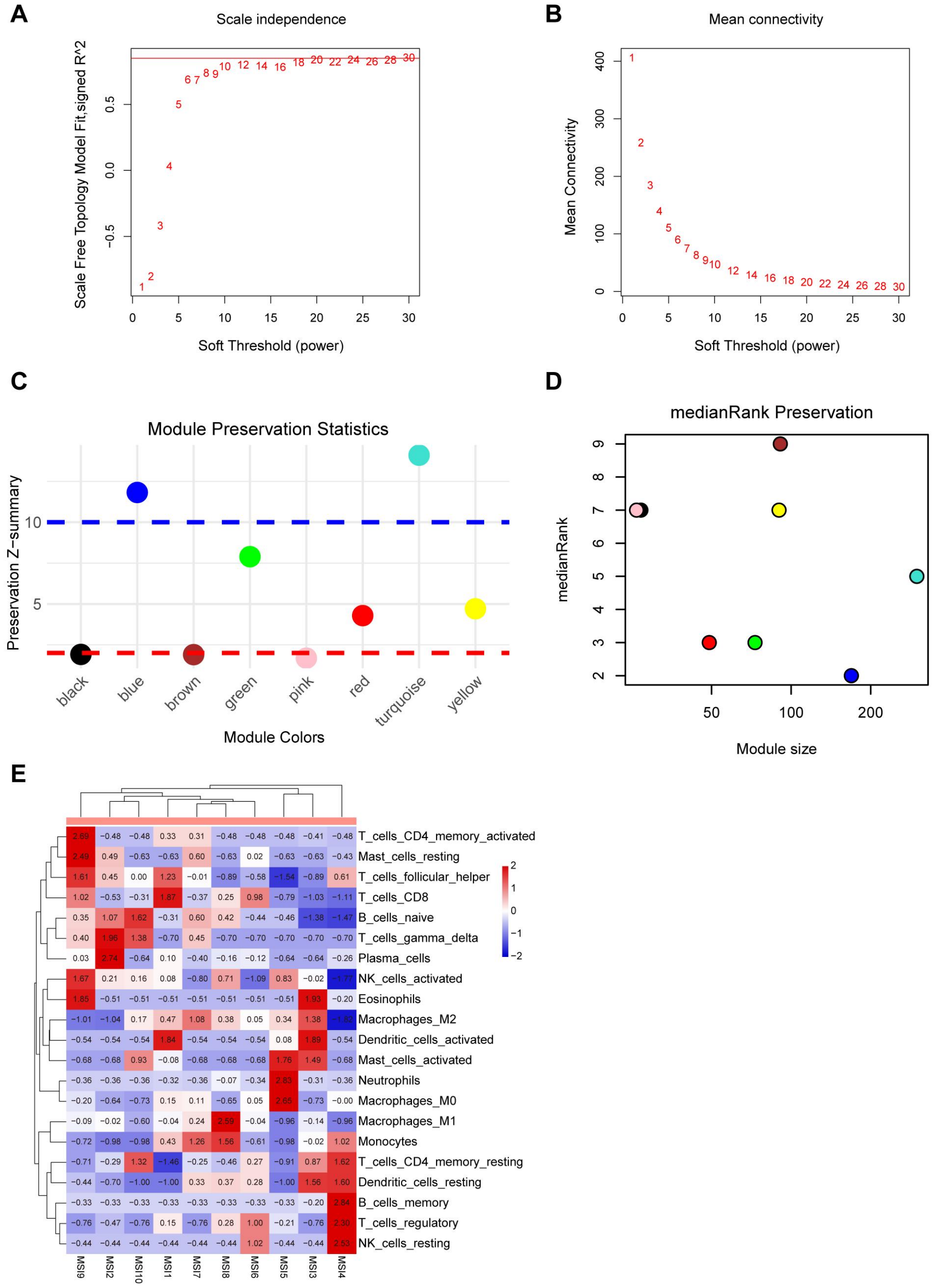

Figure S1. Identification of the circRNA module negatively associated with CD8⁺ T cell infiltration by WGCNA.

(A-B) Analysis of network topology for soft powers to identify the threshold best fit in the scale-free network. A soft power of 20 was selected to meet the threshold of R^2≥0.85. **(C-D)** Module preservation analyses of nine established modules (grey module removed). Z-summary>10 suggested strong evidence

of module preservation. Modules with Z-summary<10 were considered possibly MSI EC-specific. The higher the medianRank, the less preserved is the module relative to other modules. CircRNA modules in MSS EC samples were used as the preservation test set (not shown). **(E)** Heatmap illustrating the proportions of infiltrated immune cell subpopulations in 10 MSI EC array samples as analyzed by CIBERSORTx using mRNA data.

Figure S2

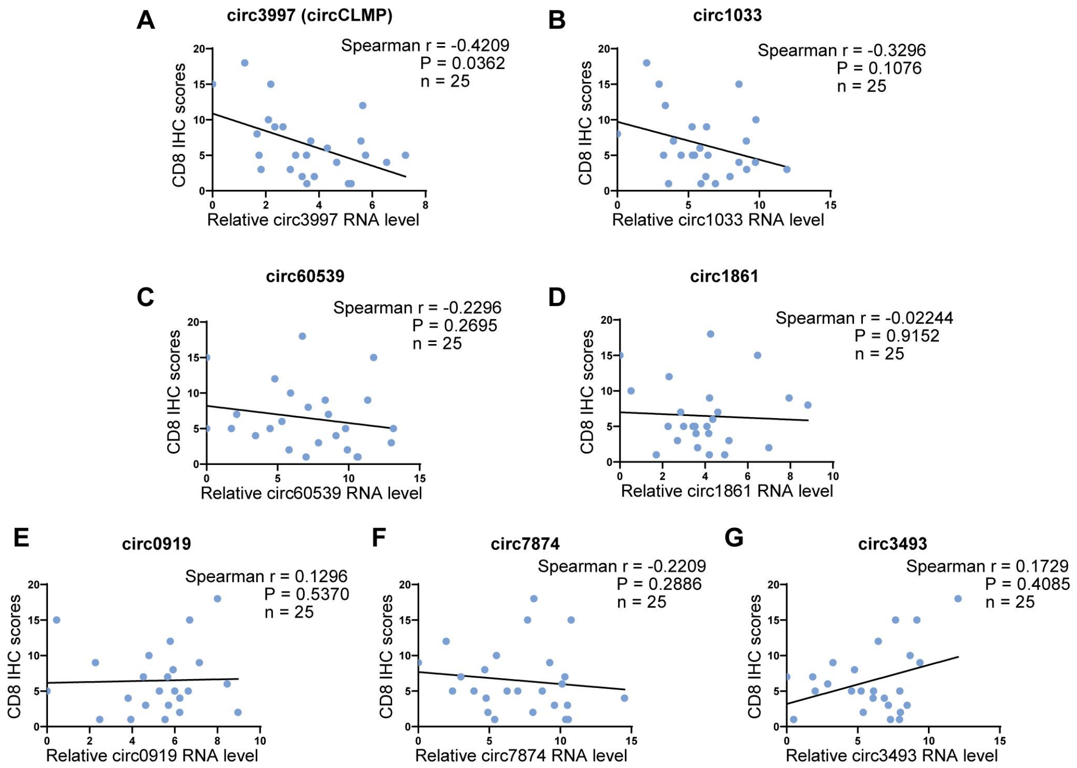

Figure S2. Correlation analyses between CD8 IHC scores and the expression level of 7 candidate circRNAs.

(A-G) Spearman's rank correlation analyses between CD8 IHC scores and the expression level of circ3997 (circCLMP), circ1033, circ60539, circ1861, circ0919, circ7874 and circ3493, respectively (n=25). Shapiro-Wilk method was used to check the normality.

Figure S3

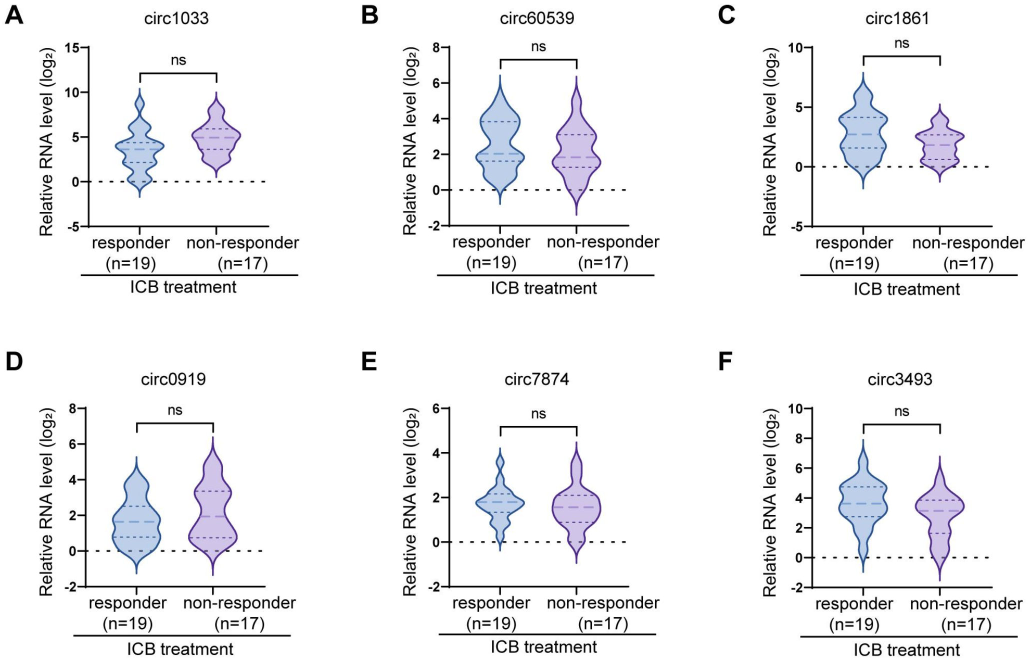

Figure S3. Expression levels of candidate circRNAs in ICB responders and ICB non-responders of MSI EC patients.

(A-F) Expression levels of circ1033, circ60539, circ1861, circ0919, circ7874, and circ3494 in ICB responders (n=19) and ICB non-responders (n=17) of MSI EC patients. Student's t test was used for significance between groups. *ns, not significant.*

Figure S4

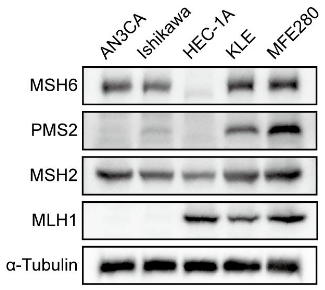

Figure S4. Western blotting detecting expressions of 4 mismatch repair proteins in EC cell lines.

KLE and MFE280 were MSS EC cells lines, while ISK and HEC-1A were MSI EC cell lines due to loss expression of any mismatch repair proteins. α-Tubulin was used as internal reference.

Figure S5

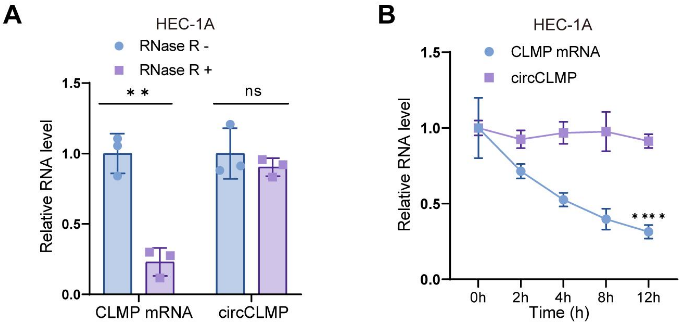

Figure S5. Characteristics of circCLMP in MSI EC cell lines.

(A) CLMP mRNA degraded in HEC-1A cells under RNase R treatment, while circCLMP showed resistance to RNase R. **(B)** CircCLMP expression in HEC-1A cells was more stable than CLMP mRNA. Each experiment was performed at least three times independently. Student' s t test was used for significance between groups. *ns, not significant; **P < 0.01; ****P < 0.0001.*

Figure S6

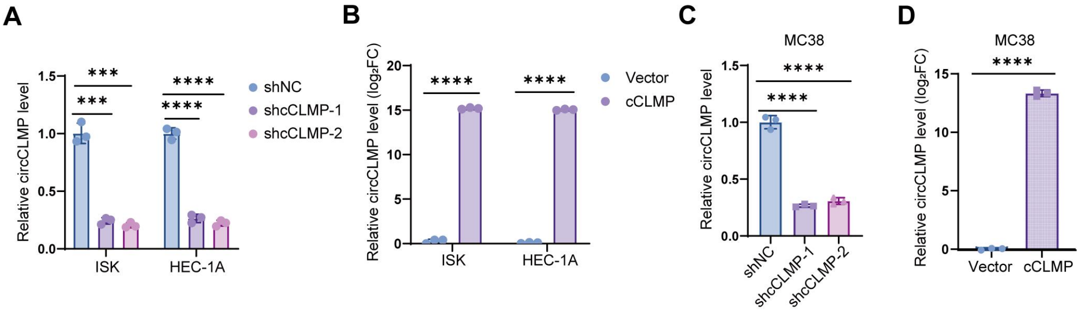

Figure S6. Validation of artificial manipulation of circCLMP.

(A-B) circCLMP was successfully silenced and overexpressed in ISK and HEC-1A as detected by RT-qPCR. **(C-D)** circCLMP was successfully silenced and overexpressed in MC38 as detected by RT-qPCR. ACTB was used as internal reference. Each experiment was performed at least three times independently. Student's t test was used for significance between groups. ****P* < 0.001; *****P* < 0.0001.

Figure S7

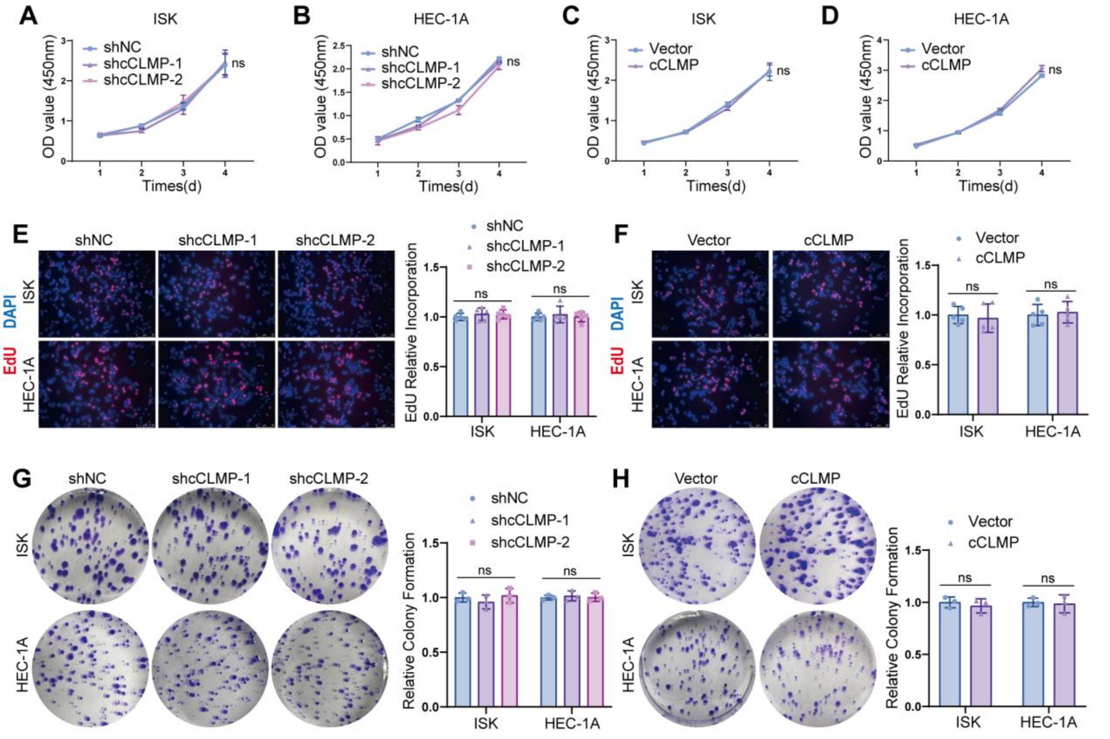

Figure S7. circCLMP has no impact on the proliferation ability of MSI EC cells.

(A-D) CCK-8 assay showed that the proliferation curves of ISK and HEC-1A were not impacted by circCLMP. **(E-F)** Inhibiting or overexpressing circCLMP in ISK and HEC-1A cells didn’t change the EdU relative incorporation. **(G-H)** Inhibiting or overexpressing circCLMP didn’t influence the colony formation of ISK and HEC-1A cells. Each experiment was performed at least three times independently. Student’s t test was used for significance between groups. ns, no significant.

Figure S8

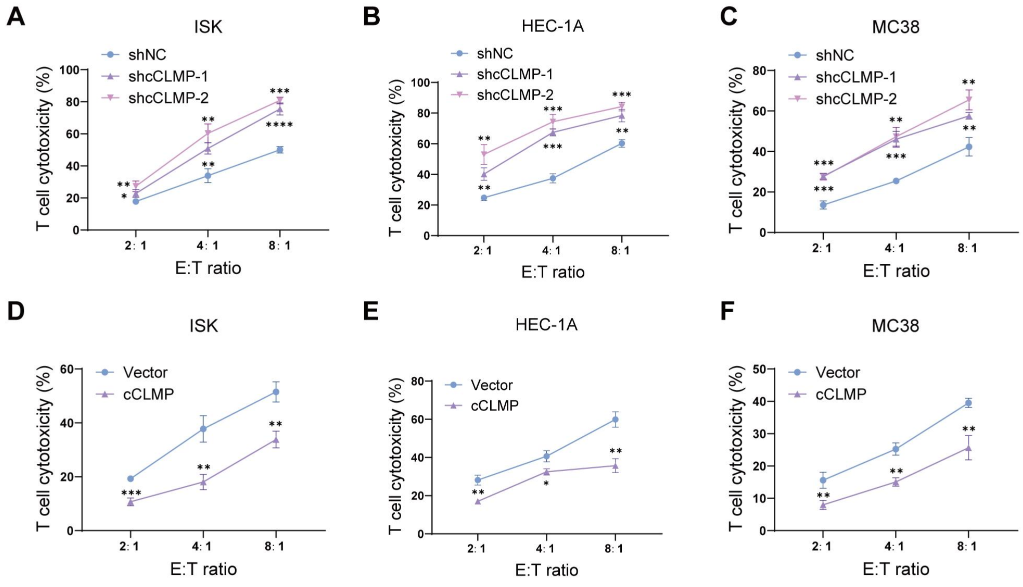

Figure S8. CircCLMP impairs killing ability of T cells.

(A-C) Inhibiting circCLMP expression markedly enhanced T cell cytotoxicity on MSI tumor cell lines. **(D-F)** Overexpressing circCLMP significantly reduced T cell-mediated MSI tumor cell lysis. Tumor cell lysis was detected by LDH release assay. Each experiment was performed at least three times independently. Student's t test was used for significance between groups. **P < 0.05; **P < 0.01; ***P < 0.001; ****P < 0.0001.*

Figure S9

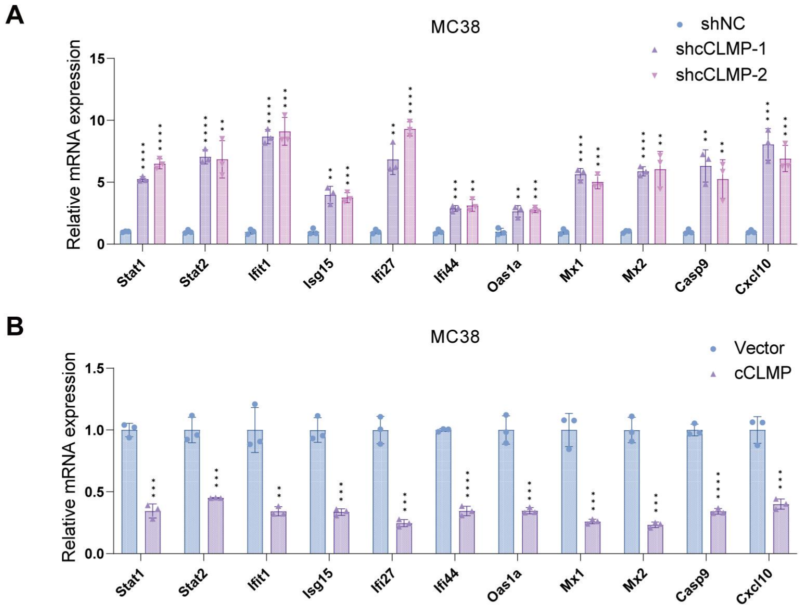

Figure S9. circCLMP suppresses interferon response in MC38.

(A) Silencing circCLMP expressions in MC38 significantly enhanced the ISGs expression. **(B)** Augmenting circCLMP expressions in MC38 significantly inhibited the ISGs expression. ACTB was used as internal reference. Each experiment was performed at least three times independently. Student's t test was used for significance between groups. ***P*<0.01; ****P* < 0.001; *****P* < 0.0001.

Figure S10

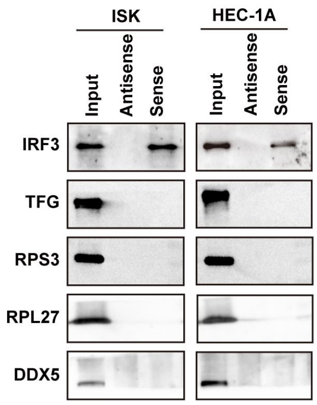

Figure S10. IRF3 is the primary interactor of circCLMP in MSI EC cells.

RNA pulldown assay showed that among the top 5 hits from mass

spectrometry results, IRF3 was detected in the proteins bound to circCLMP.

Figure S11

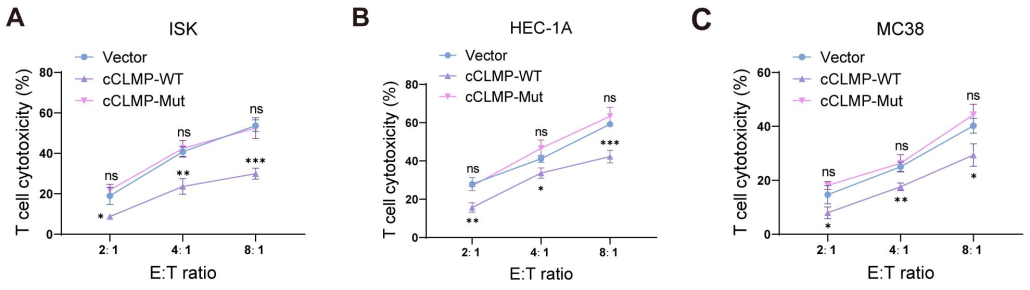

Figure S11. CircCLMP impairs killing ability of T cells in an IRF3-dependent manner.

(A-C) Overexpressing wild type circCLMP impaired T cell-mediated MSI tumor cell lysis, while enhancement of circCLMP-Mut expression had no such effects. Tumor cell lysis was detected by LDH release assay. Each experiment was performed at least three times independently. Student's t test was used for significance between groups. ns, not significant; **P < 0.05; **P < 0.01; ***P < 0.001.*

Figure S12

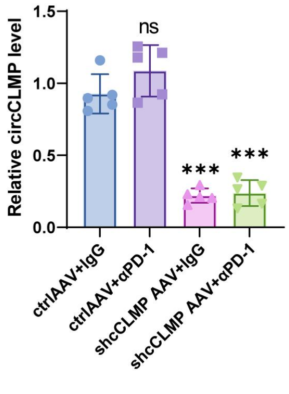

Figure S12. Transduction efficiency of AAV administration in MSI EC PDX models.

Administration of shcCLMP AAV significantly inhibited circCLMP expression in MSI EC PDX tumors (n=5 for each groups). Student's t test was used for significance between groups. ns, not significant; ****P < 0.001.*

Table S1. Sequences of primers used in this study.

**Primers for PCR and RT-qPCR**	
**Human**	
CircCLMP(divergent)-F	5'-CGAGAGAAAGAGGGAGAG-3'
CircCLMP(divergent)-R	5'-GGCTTCAGAGGTTCAATC-3'
CircCLMP(convergent)-F	5'-CACTTACTCCAGTCGTCAT-3'
CircCLMP(convergent)-R	5'-CTCCTTCCAACTCACACT-3'
Circ_0007874-F	5'-CTCAGATTGATAGGAAACTC-3'
Circ_0007874-R	5'-GCATCTACTTCCCTCATT-3'
ACTB-F	5'-CATGTACGTTGCTATCCAGGC-3'
ACTB-R	5'-CTCCTTAATGTCACGCACGAT-3'
Circ_0001861-F	5'-AGATGTCCTGACAGATAC-3'
Circ_0001861-R	5'-CTGATGACTTTGAGATTGG-3'
Circ_0003493-F	5'-AAAGTCAGTGACCGGAAT-3'
Circ_0003493-R	5'-ATGGGAAGGATGTTTGAG-3'
Circ_0060539-F	5'-CACTGTTGTCTGTTACTG-3'
Circ_0060539-R	5'-CTAATGTCCACCCTTCAA-3'
Circ_0000919-F	5'-TTCTAATCTGGCTGTGTC-3'
Circ_0000919-R	5'-CCTAACCTGAAACACACA-3'
Circ_0001033-F	5'-CCTCAGTGTGGCTAACT-3'
Circ_0001033-R	5'-TGCAGTATGAACAGCTC-3'
CLMP mRNA-F	5'-GAATGGCTGCTCACCGATAAT-3'

CLMP mRNA-R	5'-TTCAGAGGTTCAATCTGCAAGG-3'
GAPDH (convergent)-F	5'-AGAAGGCTGGGGCTCATTTG-3'
GAPDH (convergent)-R	5'-AGGGGCCATCCACAGTCTTC-3'
CDR1as-F	5'-ACGTCTCCAGTGTGCTGA-3'
CDR1as-R	5'-CTTGACACAGGTGCCATC-3'
GAPDH (divergent)-F	5'-TGTACCATCAATAAAGTACCCTGTG-3'
GAPDH (divergent)-R	5'-AAATCCGTTGACTCCGACCT-3'
U6-F	5'-ACAGATCTGTCGGTGTGGCAC-3'
U6-R	5'-GGCCCCGGATTATCCGACATTC-3'
STAT1-F	5'-CAGCTTGACTCAAAATTCCTGGA-3'
STAT1-R	5'-TGAAGATTACGCTTGCTTTTCCT-3'
STAT2-F	5'-CTGCTAGGCCGATTAACTACCC-3'
STAT2-R	5'-TCTGATGCAGGCTTTTTGCTG-3'
IFIT1-F	5'-CCCAGACTTACCTGGACAA-3'
IFIT1-R	5'-TCCTCCACACTTCAGCAA-3'
ISG15-F	5'-GGTGGACAAATGCGACGAAC-3'
ISG15-R	5'-TCGAAGGTCAGCCAGAACAG-3'
IFI27-F	5'-TGCTCTCACCTCATCAGCAGT-3'
IFI27-R	5'-CACAACTCCTCCAATCACAACT-3'
IFI44-F	5'-GGTGGGCACTAATACAACTGG-3'
IFI44-R	5'-CACACAGAATAAACGGCAGGTA-3'

OAS1-F	5'-AGTTGACTGGCGGCTATAAAC-3'
OAS1-R	5'-GTGCTTGACTAGGCGGATGAG-3'
MX1-F	5'-GTTTCCGAAGTGGACATCGCA-3'
MX1-R	5'-CTGCACAGGTTGTTCTCAGC-3'
MX2-F	5'-CACCGAGCTAGAGCTTCAGGA-3'
MX2-R	5'-CCGGGAAGGTCAATGATGGT-3'
CASP9-F	5'-CTGTCTACGGCACAGATGGAT-3'
CASP9-R	5'-GGGACTCGTCTTCAGGGGAA-3'
CXCL8-F	5'-TTTTGCCAAGGAGTGCTAAAGA-3'
CXCL8-R	5'-AACCCTCTGCACCCAGTTTTC-3'
CXCL10-F	5'-ACTGCCATTCTGATTTGCTGC-3'
CXCL10-R	5'-ATGCAGGTACAGCGTACAGT-3'
**Mouse**	
CircCLMP-F	5'-GTATTATTGGCAGCGAATC-3'
CircCLMP-R	5'-CTCGGTCAAGTTATTGTAGA-3'
Stat1-F	5'-TCACAGTGGTTCGAGCTTCAG-3'
Stat1-R	5'-GCAAACGAGACATCATAGGCA-3'
Stat2-F	5'-TCCTGCCAATGGACGTTCG-3'
Stat2-R	5'-GTCCCACTGGTTCAGTTGGT-3'
Ifit1-F	5'-TTCCGTAGGAAACATCGCGT-3'
Ifit1-R	5'-ACATTGTCCTGCCTTCTGGG-3'

Isg15-F	5'-GGTGTCCGTGACTAACTCCAT-3'
Isg15-R	5'-TGGAAAGGGTAAGACCGTCCT-3'
Ifi27-F	5'-CTAAGATGATGTCCTTGTC-3'
Ifi27-R	5'-TCCTTCTCTGTGTTAGAC-3'
Ifi44-F	5'-AACTGACTGCTCGCAATAATGT-3'
Ifi44-R	5'-GTAACACAGCAATGCCTCTTGT-3'
Oas1a-F	5'-CTGGACAAGTTCATAGAG-3'
Oas1a-R	5'-CTGGTGAGATTGTTAAGG-3'
Mx1-F	5'-GACCATAGGGGTCTTGACCAA-3'
Mx1-R	5'-AGACTTGCTCTTTCTGAAAAGCC-3'
Mx2-F	5'-GAGGCTCTTCAGAATGAGCAAA-3'
Mx2-R	5'-CTCTGCGGTCAGTCTCTCT-3'
Casp9-F	5'-GACGCTCTGCTGAGTCGAG-3'
Casp9-R	5'-GGTCTAGGGGTTTAACAGCCTC-3'
Cxcl10-F	5'-CCAAGTGCTGCCGTCATTTTC-3'
Cxcl10-R	5'-GGCTCGCAGGGATGATTTCAA-3'
Actb-F	5'-CCCCTGAACCCTAAGGCCA-3'
Actb-R	5'-CGGACTCATCGTACTCCTGC-3'
**Primers for in vitro transcription**	
circCLMP(1-493)-F	5'-TAATACGACTCACTATAGGGGTGATCACTTACTCCAG-3'
circCLMP(1-493)-R	5'-ACTGTACAGTTACTCGCACCACACA-3'

circCLMP(1-152)-F	5'-TAATACGACTCACTATAGGGGTGATCACTTACTCCAG-3'
circCLMP(1-152)-R	5'-ACCTTACAGGTGTACCGGCCCT-3'
circCLMP(1-96)-F	5'-TAATACGACTCACTATAGGGGTGATCACTTACTCCAG-3'
circCLMP(1-96)-R	5'-GGCATCTCCTGCCAGGAAATTGGA-3'
circCLMP(97-152)-F	5'-TAATACGACTCACTATAGGGTCCTTGCAGATTGAACCT-3'
circCLMP(97-152)-R	5'-ACCTTACAGGTGTACCGGCCCT-3'
circCLMP(153-290)-F	5'-TAATACGACTCACTATAGGGTAAGAATTCAGGGCGCTA-3'
circCLMP(153-290)-R	5'-GTGCCAGAGGATGACTCACACTGC-3'
circCLMP(291-370)-F	5'-TAATACGACTCACTATAGGGAGAGCCCATTGTGTATTACT-3'
circCLMP(291-370)-R	5'-CAATCCTAGATTTGGGAGGCAGACG-3'
circCLMP(371-493)-F	5'-TAATACGACTCACTATAGGGACTACAACCACCCTGGA-3'
circCLMP(371-493)-R	5'-ACTGTACAGTTACTCGCACCACACA-3'
circCLMP(antisense)-F	5'-TAATACGACTCACTATAGGGACTGTACAGTTACTCGCACCACACA-3'
circCLMP(antisense)-R	5'-GTGATCACTTACTCCAGTCGTCAT-3'

Table S2. shRNAs used in this study.

**shRNAs**	**Sequences**
shcCLMP-1 (human)	Sense 5'-GUAACUGUACAGUGUGAUCAC-3'
shcCLMP-2 (human)	Sense 5'-AACUGUACAGUGUGAUCACUU-3'
shcCLMP-1 (mouse)	Sense 5'-GUGACUGUACAGUGUUAUUAC-3'
shcCLMP-2 (mouse)	Sense 5'-GACUGUACAGUGUUAUUACGU-3'

Table S3. Antibodies used for flow cytometry in the study.

Panel	CAS	channel
CD45	Biolegend, #103116	APC
CD4	Biolegend, #100422	PE/Cy7
CD25	Biolegend, #101908	FITC
FOXP3	Biolegend, #126419	BV421
CD3	Biolegend, #100204	FITC
CD8a	Biolegend, #100742	BV650
Fixable Viability Dye	Biolegend, #423105	
TruStain FcX (anti-mouse CD16/32)	Biolegend, #101320	

Table S4. Primary antibodies used for IF, IHC and WB in the study.

Target protein	Antibody	Application
CD8 (Human)	ZSGB-BIO, #ZA-0508	IHC (1:1000)
IRF3	Proteintech, #11312-1-AP	WB (1:10000); IP (2 μg for 1.0 mg of total protein); IF (1:200)
pIRF3-S386	CST, #37829T	WB (1:1000)
pIRF3-S386	Huabio, #ET1608-22	WB (1:1000)
pIRF3-S396	CST, #4947T	WB (1:1000)
TBK1	Proteintech, # 28397-1-AP	WB (1:1000)
GAPDH	Proteintech, #10494-1-AP	WB (1:10000)
H3	Merck Millipore, #06-755	WB (1:2000)
CD8a (Mouse)	Abcam, #ab316778	IF (1:100)
α-Tubulin	Proteintech, #80762-1-RR	WB (1:10000)
MLH1	Proteintech, #11697-1-AP	WB (1:2000)
MSH2	Proteintech, #15520-1-AP	WB (1:1000)

MSH6	Proteintech, #18120-1-AP	WB (1:5000)
PMS2	Proteintech, #18164-1-AP	WB (1:5000)
TFG	Proteintech, #11571-1-AP	WB (1:1000)
RPS3	Proteintech, #11990-1-AP	WB (1:5000)
RPL27	Proteintech, #14980-1-AP	WB (1:1000)
DDX5	Proteintech, #10804-1-AP	WB (1:1000)

Table S5. List of mass-spectrometry hits.

rank	prot_acc	prot_hit_num	prot_score	prot_mass	emPAI
1	sp|Q14653|IRF3_HUMAN	59	97	27842	0.97
2	sp|Q92734|TFG_HUMAN	23	71	43478	0.4
3	sp|P23396|RPS3_HUMAN	39	47	26842	0.26
4	sp|P61353|RPL27_HUMAN	82	23	15788	0.22
5	sp|P17844|DDX5_HUMAN	18	101	69618	0.2
6	sp|P28340|DPOD1_HUMAN	8	233	125035	0.15
7	sp|P0DMV8|HS71A_HUMAN	31	57	70294	0.15
8	sp|P11940|PABP1_HUMAN	37	50	70854	0.15
9	sp|P46781|RS9_HUMAN	56	36	22635	0.15
10	sp|P31943|HNRH1_HUMAN	33	55	49484	0.14
11	sp|Q92841|DDX17_HUMAN	16	112	80906	0.13
12	sp|P35637|FUS_HUMAN	34	51	53622	0.13
13	sp|P62906|RL10A_HUMAN	85	22	24987	0.13
14	sp|P14618|KPYM_HUMAN	46	42	58470	0.12
15	sp|P31275|HXC12_HUMAN	63	32	30437	0.11
16	sp|Q6IPR3|TYW3_HUMAN	78	26	30231	0.11
17	sp|Q9BRL6|SRSF8_HUMAN	51	37	32382	0.1

18	sp|Q96HS1|PGAM5_HUMAN	54	37	32213	0.1
19	sp|Q9NWB6|ARGL1_HUMAN	72	27	33197	0.1
20	sp|Q6ZMR3|LDH6A_HUMAN	40	46	36826	0.09
21	sp|Q93070|NAR4_HUMAN	64	31	36198	0.09
22	sp|P08238|HS90B_HUMAN	30	58	83554	0.08
23	sp|Q13247|SRSF6_HUMAN	48	40	39677	0.08
24	sp|Q9BYG4|PAR6G_HUMAN	65	31	41086	0.08
25	sp|Q9BVA1|TBB2B_HUMAN	61	33	50377	0.07
26	sp|P00966|ASSY_HUMAN	73	27	46786	0.07
27	sp|P0DPH7|TBA3C_HUMAN	79	25	50612	0.07
28	sp|Q8N684|CPSF7_HUMAN	60	33	52189	0.06
29	sp|Q01844|EWS_HUMAN	55	36	68721	0.05
30	sp|Q7Z417|NUFP2_HUMAN	70	27	76132	0.04
31	sp|Q6NUI2|GPAT2_HUMAN	87	14	89205	0.04
32	sp|P78332|RBM6_HUMAN	74	27	129192	0.03
33	sp|Q5VYS8|TUT7_HUMAN	77	26	173288	0.02
34	sp|Q8TF72|SHRM3_HUMAN	62	33	218321	0.01
35	sp|Q9Y2I7|FYV1_HUMAN	76	26	239609	0.01
36	sp|Q9UQ35|SRRM2_HUMAN	80	24	300179	0.01
